# Circulating miR-122-5p, miR-151a-3p, miR-126-5p and miR-21-5p as potential predictive biomarkers for Metabolic Dysfunction-Associated Steatotic Liver Disease assessment

**DOI:** 10.1007/s13105-024-01037-8

**Published:** 2024-08-14

**Authors:** Ana Luz Tobaruela-Resola, Fermín I. Milagro, Mariana Elorz, Alberto Benito-Boillos, José I. Herrero, Paola Mogna-Peláez, Josep A. Tur, J. Alfredo Martínez, Itziar Abete, M. Ángeles Zulet

**Affiliations:** 1 Department of Nutrition, Food Sciences and Physiology and Centre for Nutrition Research, Faculty of Pharmacy and Nutrition, University of Navarra, 31008 Pamplona, Spain; 2 Navarra Institute for Health Research (IdiSNA), 31008 Pamplona, Spain; 3 Biomedical Research Centre Network in Physiopathology of Obesity and Nutrition (CIBERobn), Instituto de Salud Carlos III, 28029 Madrid, Spain; 4 Department of Radiology, Clínica Universidad de Navarra, 31008 Pamplona, Spain; 5 Liver Unit, Clínica Universidad de Navarra, 31008 Pamplona, Spain; 6 Biomedical Research Centre Network in Hepatic and Digestive Diseases (CIBERehd), 28029 Madrid, Spain; 7 Research Group On Community Nutrition and Oxidative Stress, University of Balearic Islands, 07122 Palma, Spain; 8 Precision Nutrition and Cardiovascular Health Program, IMDEA Food, CEI UAM + CSIC, Madrid, Spain

**Keywords:** miRNA, MASLD, Biomarker, Steatosis, Liver stiffness, Hepatic fat

## Abstract

**Abstract:**

Metabolic Dysfunction-Associated Steatotic Liver Disease (MASLD) is a worldwide leading cause of liver-related associated morbidities and mortality. Currently, there is a lack of reliable non-invasive biomarkers for an accurate of MASLD. Hence, this study aimed to evidence the functional role of miRNAs as potential biomarkers for MASLD assessment. Data from 55 participants with steatosis (MASLD group) and 45 without steatosis (control group) from the Fatty Liver in Obesity (FLiO) Study (NCT03183193) were analyzed. Anthropometrics and body composition, biochemical and inflammatory markers, lifestyle factors and liver status were evaluated. Circulating miRNA levels were measured by RT-PCR. Circulating levels of miR-122-5p, miR-151a-3p, miR-126-5p and miR-21-5p were significantly increased in the MASLD group. These miRNAs were significantly associated with steatosis, liver stiffness and hepatic fat content. Logistic regression analyses revealed that miR-151a-3p or miR-21-5p in combination with leptin showed a significant diagnostic accuracy for liver stiffness obtaining an area under the curve (AUC) of 0.76 as well as miR-151a-3p in combination with glucose for hepatic fat content an AUC of 0.81. The best predictor value for steatosis was obtained by combining miR-126-5p with leptin, presenting an AUC of 0.95. Circulating miRNAs could be used as a non-invasive biomarkers for evaluating steatosis, liver stiffness and hepatic fat content, which are crucial in determining MASLD.

**Clinical trial registration:**

• Trial registration number: NCT03183193 (www.clinicaltrials.gov).

• Date of registration: 12/06/2017.

**Supplementary Information:**

The online version contains supplementary material available at 10.1007/s13105-024-01037-8.

## Introduction

One of the leading causes of liver-related associated morbidities and mortality is non-alcoholic fatty liver disease (NAFLD), which is characterized by an excess storage of triglycerides (TG) and fatty acids in the hepatocytes [55]. In recent years, the incidence of this disease has increasing, reaching a global prevalence about 30% [63]. This disease encompasses a wide range of histopathology that goes from simple hepatic steatosis to non-alcoholic steatohepatitis (NASH), and can reach into fibrosis, cirrhosis and cellular hepatocarcinoma (HCC) [55].

In June 2023, a multi-society Delphi consensus statement introduced a new terminology, replacing NAFLD and Metabolic Dysfunction Associated Fatty Liver Disease (MAFLD) with Metabolic Dysfunction-Associated Steatotic Liver Disease (MASLD). Similarly, NASH was replaced with Metabolic Dysfunction-Associated Steatohepatitis (MASH). These new terms encompass the presence of at least one to five cardiometabolic risk factors in their diagnostic criteria [51]. Even with the revised definition, about 99% of patients previously diagnosed with NAFLD meet the diagnostic criteria for MASLD [28].

Due to interactions among genetic, metabolic, and environmental factors, there is an interindividual variation in the MASLD phenotype and its progression, which currently remain unclear [66]. MASLD is strongly associated with chronic liver disease, cardiovascular disease (CVD), type 2 diabetes mellitus (T2DM), obesity, insulin resistance (IR), hypertension, hyperlipidemia, and metabolic syndrome [55, 62].

Liver biopsy continues to be the reference technique for the diagnosis and prognosis of the disease. However, it carries a risk for the patient because it is an invasive procedure, and it can cause complications, as well as errors in the sample [30]. Imaging modalities such as ultrasonography (US), computed tomography (CT), and magnetic resonance imaging (MRI) provide non-invasive options for detecting MASLD. However, these techniques are frequently associated with significant time consumption and high costs [57]. There is a need for improved non-invasive tools that facilitate the diagnosis and staging of MASLD, along with the development of methods to identify those at risk of disease progression [30].

MASLD involves many signaling molecules contributing to hepatic metabolism, inflammatory, oxidative, and fibrotic processes, making it quite complex [45]. These include microRNAs (miRNAs), which are small non-coding RNAs that play important roles in the regulatory processes of gene expression and target numerous genes involved in the glucose and lipid metabolism, inflammation, cell proliferation, apoptosis and necrosis, in turn involved in the pathogenesis of MASLD [10, 11].

Although it is not fully understanding the exact molecular drivers and biological pathways responsible for disease progression, genetic variations likely play a role in the complexity and differences observed in the disease phenotype [48]. Alteration in circulating miRNAs play an important role in hepatocyte function, liver injury, viral hepatitis, ALD, MASLD, liver fibrosis progression, and HCC [43]. Moreover, miRNAs regulate multiple signaling pathways involved in lipid accumulation, IR, oxidative stress, and inflammatory responses, thereby contributing to the development and advancement of MASLD [37]. MiR-122, a key liver-specific miRNA, plays a role regulating liver metabolism and maintaining fatty acid balance, previously correlated with steatosis severity in MASLD [5], playing an important role in MASLD pathogenesis as well as miR-21 and miR-192 [60]. In addition, miR-126-5p could impact on pathogenesis of liver fibrosis and it is associated with T2DM, obesity, metabolic syndrome [4], adiposity, lipid and glucose metabolism and inflammation as well as miR-222 [8]. Additionally, miR-151 which is linked to TNF-α [40], and miR-15b-3p and miR-29b-3p, which are especially related with high expression in hepatic stellate cells, have been associated with liver tumor development, suggesting their involvement in hepatic lesions [31, 60].

Despite some associations between those miRNAs and MASLD, a comprehensive understanding of the complex involvement of miRNAs in the disease is needed. In this context, the aim of the present study was examining the functional role of miRNAs as hepatic status biomarkers using simple samples such as serum for the diagnosis and management of MASLD.

## Material and methods

### Study participants

This research is a cross-sectional analysis that include the evaluation of baseline measurements of 55 subjects with overweight or obesity (BMI ≥ 27.5 kg/m^2^ < 40 kg/m^2^) with MASLD (diagnosed by ultrasonography) obtained from a randomized controlled trial, the FLiO (Fatty liver in obesity) study (www.clinicaltrials.gov; NCT03183193), and 45 subjects with normal-weight (BMI < 25 kg/m^2^) without MASLD (confirmed by ultrasonography) obtained from the EHGNA study (a FLiO study continuation). From them, miRNAs circulating levels have been obtained. The exclusion criteria have already been described previously [42].

The study protocol was approved by the Research Ethics Committee of the University of Navarra (ref. 54/2015). Everyone gave written informed consent before enrolling in the study. All procedures were performed in compliance with significant national regulations, institutional policies, and in accordance with the Declaration of Helsinki and following the CONSORT 2010 guidelines.

### Anthropometric and body composition evaluation

Anthropometric measurements (BW and waist circumference) and body composition (Lunar iDXA, enCORE 14.5, Madison, WI) were determined under fasting conditions in the Nutritional Intervention Unit of the University of Navarra, according to standardized procedures [14]. Body Mass Index (BMI) was calculated as BW divided by height squared (Kg/m^2^).

### Biochemical determinations

Blood samples were collected, processed and stored at -80 degrees, until further analysis [18]. Biochemical determinations such as blood glucose, glycosylated hemoglobin (HbA1c), triglycerides (TG), total cholesterol (TC), high-density lipoprotein cholesterol (HDL-c), alanine aminotransferase (ALT), aspartate aminotransferase (AST) and gamma glutamyl transferase (GGT) were measured in a Pentra C-200 autoanalyzer (HORIBA ABX, Madrid, Spain) Spain) with specific commercial kits and following the manufacturer’s instructions (Cobas 8000, Roche Diagnostics, Switzerland). Low-density lipoprotein (LDL-c) levels were calculated using the Friedewald formula [25]: LDL-c = TC—HDL-c—TG/5. The Castelli’s Risk Index (CRI) was calculated with the formula: Total cholesterol/HDL-c, as previously described [23]. The fatty liver index (FLI) was calculated using serum TG, BMI, waist circumference, and GGT concentrations, as mentioned [6]. FLI values < 30 rule out hepatic steatosis, and values ≥ 60 indicate hepatic steatosis. Concentrations of insulin, leptin, chemerin, retinol-binding protein (RBP4) and adiponectin were measured using ELISA kits (Demeditec; Kiel-Wellsee, Germany) in a Triturus autoanalyzer (Grifols, Barcelona, Spain). Leukocyte cell-derived chemotaxin-2 (LECT2) was analyzed using the Triturus Autoanalyzer (Grifols, Barcelona, Spain) with specific kits (Biovendor LLC, North Carolina, United States). Insulin resistance was estimated through the Homeostasis Model Assessment Index (HOMA-IR), which was calculated using a previously described formula [14].

### Imaging techniques in the evaluation of liver status

The liver status assessment was carried out under fasting conditions at the University of Navarra Clinic by highly qualified personnel. To determine the presence of hepatic steatosis, ultrasonography was performed (Siemens ACUSON S2000 and S3000), according to the methodology already described [9]. To determine liver fat content and hepatic volume, magnetic resonance was used (Siemens Aera 1.5 T, Erlangen Germany), applying the DIXON technique [9]. Multi-echo T2 corrected single breath-hold spectroscopy (Histo) of a single voxel and multi-echo 3D gradient echo imaging with Dixon reconstruction and T2 correction were included by quantitative sequences [18]. ARFI elastography was performed to determine liver stiffness, using the value obtained from 10 valid ARFI measurements of each subject as the mean value [9].

### Dietary intervention and lifestyle

Dietary intake was assessed with a semiquantitative food frequency questionnaire (FFQ) with 173 items, validated in Spain for daily energy and nutrient intake [22]. Daily food consumption was estimated by multiplying the portion size by the consumption frequency and dividing as described elsewhere [46]. The nutrient composition of the specified serving size for each food was estimated using Spanish food composition tables [46]. Adherence to the Mediterranean diet (MedDiet) was assessed with a 17-item screening questionnaire, with a final score ranging from 0 to 17, with a higher score indicating better MedDiet adherence [26]. Physical activity was estimated using a validated Spanish version of Minnesota leisure-Time Physical Activity Questionnaire [42]. The physical activity was classified into four different categories (sedentary, light, moderate or vigorous) based on the International Physical Activity Questionnaire (IPAQ) [41].

### RNA isolation, reverse transcription and Real-Time PCR (RT-PCR)

Serum was isolated from whole blood by centrifugation at 1100 g at 4 °C for 15 min (Modelo5415R, Eppendorf AG, Hamburgo, Germany) and then, samples were frozen (-80 ºC) until RNA reverse transcription. After RNA extraction, total RNA of the serum sample was isolated with RNeasy Serum/Plasma Advanced Kit (Qiagen, Hilden, Germany) following manufacturer’s instructions. For the procedure, absolute ethanol (1L) and Isopropanol (2-Propanol, gradient HPLC grade) were used. cDNA was synthesized using 4 μl of miRNA sample, miRCURY LNA RT Kit (Qiagen, Hilden, Germany), which allowed the detection of the miRNAs of interest. Once the reverse transcription was realized, the protocol for the expression of miRNA isolated from serum was carried out. Quantitative PCR (qPCR) was performed with the CFX384 Touch Real-Time PCR system (Bio-Rad, Hercules, CA, USA) using the miRCURY SYBR® Green PCR Kit (Qiagen, Hilden, Germany). Finally, miRNAs Relative Quantities (RQs) were calculated with the formula *2*^*−*ΔCt^. Normalization factor (NF) was calculated using the geometric mean of RQs of all expressed miRNAs per sample and the normalized relative quantities were obtained dividing RQs by the sample specific NF as previously described [44]. The values were expressed as fold change (FC) of each miRNA with respect to the exogenous reference gene Unisp6 [44], the spike-in used to assess the quality of the cDNA synthesis and qPCR process.

### *In Silico *evaluation

Based on scientific literature, we selected the miRNAs that have been previously associated with MASLD and its comorbidities [12, 35, 66]. The association between miRNAs, validated target genes and diseases focusing on those miRNAs showing significant differences between control and intervention groups were analyzed. Once the relevant miRNAs were identified, we linked them to previously validated genes in humans by utilizing all publicly available database through miRWalk (http://mirwalk.umm.uni-heidelberg.de/; accessed on 11/06/2023). Among the genes associated with our miRNAs, we selected those with significant results. Subsequently, we specifically chose genes associated with MASLD or fatty liver disease, relying on the data provided by the DisGeNet (https://www.disgenet.org; accessed on 26/06/2023) database. To investigate interactions between the selected miRNAs and their target genes a network was created using miRNet (https://www.mirnet.ca/accessed on 28/06/2024). We amplified and highlighted in black those target genes that are shared between our specific miRNAs, which were marked in blue. The genes were obtained through miRTaBase v8.0 and TarBase v8.0 (ID type miRbase). Furthermore, to discover metabolic pathways and biological process from validated target genes associated with each miRNA, we used GeneCodis (https://genecodis.genyo.es/; accessed on 28/06/2024), choosing *homo sapiens* as a main organism.

### Statistical analyses

The normality of the variables was evaluated using Shapiro–wilk test. Data presentation depended on distribution (mean ± standard deviation (SD) or median ± interquartile range (IQR)). Differences between groups were assessed using the Student’s t test or the Mann–Whitney U test and categorical variables with X2 test. Differences between miRNAs were presented as median ± (SE). The association between variables was evaluated using the Pearson correlation coefficient or Spearman's rho (p), as appropriate.

Univariate and multivariate logistic regressions analyses were performed with liver steatosis, liver stiffness or hepatic fat content variables as dependent variables and miRNAs and other covariables as independent variables. Receiver Operating Characteristic Curve Analyses (ROC) and the areas under the ROC curve (AUROC) were calculated to assess the predictive power of circulating miRNAs and their combinations with hepatic steatosis degree, liver stiffness and hepatic fat content. Regression models were adjusted by potential cofounders including sex, age, physical activity and inflammatory and biochemical markers related to MASLD (adiponectin, RBP4, TG, glucose, LECT2, leptin and chemerin). For the regressions analyses, steatosis degree was categorized into two groups (without steatosis vs mild, moderate and severe steatosis) and liver stiffness (m/s) (≤ 1.31 vs > 1.31) and hepatic fat content (%) (≤ 4.4 vs > 4.4) according to the median. Optimism-corrected value was used to validate the results obtained using the Tibshirani´s enhanced bootstrap method described previously [29].

Statistical analyses were carried out with the statistical program Stata version 15.0 (StataCorp 2011, College Station, TX, USA). All p values presented were two-tailed. Differences were considered statistically significant when p < 0.05.

### Generative *IA* and *IA*-assisted technologies in the writing process

During the preparation of this work the author used OpenAI in order to enhance readability and language of the manuscript. After using this tool, the author reviewed and edited the content as needed and take full responsibility for the content of the publication.

## Results

### Overview of baseline characteristics

Characteristics of MASLD (n = 55) and control (n = 45) groups are shown in Table 1. Significant differences were found between both groups in body composition, anthropometric measures and biochemical determinations, clearly proving worse health status in MASLD group (Supplementary Fig. 1). Hepatic transaminases such as ALT and GGT, hepatic fat content, hepatic volume and steatosis degree were significantly increased in MASLD (p < 0.001). LECT2, chemerin, RBP4 and leptin were significantly increased in MASLD group, nevertheless adiponectin was significantly lower (p < 0.001). Lipid profile including total cholesterol, HDL and LDL was significantly lower in MASLD group than controls, although CRI was significantly higher in MASLD group as well as TG. MedDiet adherence score was significantly lower in MASLD group compared to controls (p < 0.001). No significant differences were found in physical activity between both groups.

**Table 1:** Body composition, biochemical determinations, lifestyle parameters, inflammatory markers and hepatic status of the Control and MASLD groups of the study

	Control group (n = 45)	MASLD group (n = 55)	P-value
Body composition and lifestyle markers			
Weight (kg)	65.6 (12.0)	94.6 (14.5)	< 0.001
BMI (kg/m^2)^	23.1 (21.5–24.9)	32.3 (30.2–35.8)	< 0.001
VAT (kg)	0.2 (0.1–0.7)	2.1 (1.6–2.7)	< 0.001
Body fat (%)	31.7 (27.0–37.4)	40.7 (36.3–45.9)	< 0.001
Waist circumference (cm)	78.1 (71.3–85.5)	108.7 (102.0–116.0)	< 0.001
Fat mass (Kg)	19.3 (5.7)	37.8 (8.8)	< 0.001
MedDiet adherence score	9.9 (2.4)	5.8 (2.3)	< 0.001
Total energy (kcal/day)	2155 (1759–2607)	2590 (2154 -2895)	0.008
Physical Activity	1.26 (1.0)	1.03 (1.0)	0.265
Hepatic status			
Steatosis degree	0 (0–0)	1 (1–2)	< 0.001
Hepatic fat content (%)	2.9 (2.4–3.4)	9.2 (5.7–13.9)	< 0.001
Liver vol. (ml)	1276.0 (1127.0–1494.5)	1697.0 (1409.0–2002.0)	< 0.001
Liver stiffness (m/s)	1.1 (1.0–1.4)	1.5 (1.1–2.3)	< 0.001
FLI index	8.4 (5.1–21.3)	84.5 (73.7–92.3)	< 0.001
ALT (IU/L)	16.2 (12.5–26.8)	30.0 (21.0–43.0)	< 0.001
AST (IU/L)	20.9 (16.9–26.8)	24.0 (19.0–28.0)	0.110
GGT (IU/L)	17.0 (12.0–24.0)	30.0 (20.0–44.0)	< 0.001
Biochemical determinations and inflammatory markers			
Glucose (mg/dL)	89.3 (85.8–96.1)	102.0 (92.0–109.0)	< 0.001
Insulin (mU/L)	4.2 (2.7–5.0)	16.3 (12.0–20.9)	< 0.001
HbA1c (%)	5.1 (5.0–5.3)	5.6 (5.4–5.9)	< 0.001
HOMA-IR	0.9 (0.6–1.1)	4.2 (2.9–5.7)	< 0.001
TC (mg/dL)	218.0 (40.2)	188.7 (37.0)	< 0.001
HDL-c (mg/dL)	65.7 (53.1–83.8)	51.0 (41.0–60.0)	< 0.001
LDL-c (mg/dL)	133.5 (32.3)	110.0 (33.1)	< 0.001
CRI (mg/dL)	3.1 (2.6–3.7)	3.54 (3.0–4.2)	0.029
Triglycerides (mg/dL)	73.0 (58.0–93.0)	123.0 (86.0–150.0)	< 0.001
Chemerin (ng/ml)	174.0 (151.0–214.0)	207.0 (191.0–230.0)	< 0.001
LECT2 (ng/ml)	23.8 (9.0)	41.3 (10.5)	< 0.001
RBP4 (mg/L)	26.9 (23.3–30.4)	33.0 (28.2–41.6)	< 0.001
Leptin (ng/mL)	11.6 (4.0–21.7)	26.8 (15.8–41.4)	< 0.001
Adiponectin (μg/mL)	13.1 (9.7–18.6)	6.6 (5.6–8.9)	< 0.001

Values are expressed as mean (SD) or median (IQR), according to the data distribution. Abbreviations: MASLD, Metabolic Dysfunction-Associated Steatotic Liver Disease; BMI, Body Mass Index; VAT, Visceral Adipose Tissue; MedDiet, Mediterranean Diet; FLI, Fatty Liver Index; ALT, Alanine aminotransferase; AST, Aspartate aminotransferase; GGT, Gamma-glutamyl transferase; HbA1C, Hemoglobin A1C; HOMA-IR, Homeostatic model Assessment for Insulin Resistance; TC, Total Cholesterol; HDL-c, High Density Lipoprotein cholesterol; LDL-c, Low Density Lipoprotein cholesterol; CRI, Castelli Risk Index; LECT2, Leukocyte cell-derived chemotaxin-2; RBP4, Retinol Binding Protein

### Analyses of circulating miRNAs levels and their associations with MASLD and metabolic factors

**Fig. 1:**
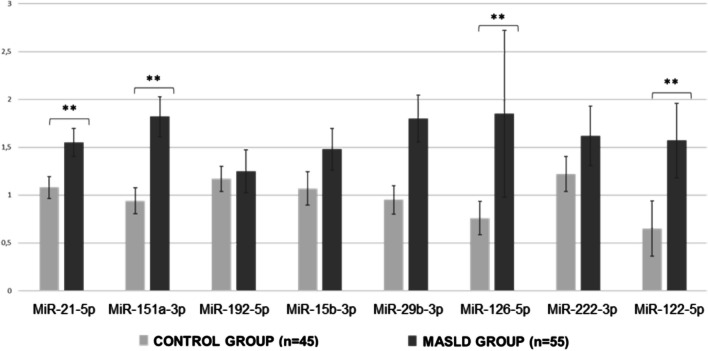
Differences in circulating miRNAs levels between MASLD and control group. Values are expressed as median (SE). Abbreviations: MASLD, Metabolic Dysfunction-Associated Steatotic Liver Disease; miRNA, microRNA. P-value: p < 0.01**

Eight miRNAs related with MASLD were selected and its circulating levels were analyzed (miR-21-5p, miR-151a-3p, miR-192-5p, miR-15b-3p, miR-29b-3p, miR-126-5p, miR-222-3p and miR-122-5p). Only circulating miR-122-5p, miR-151a-3p, miR-126-5p and miR-21-5p levels were significantly higher in MASLD group in comparison with control group (Fig. 1).

**Fig. 2:**
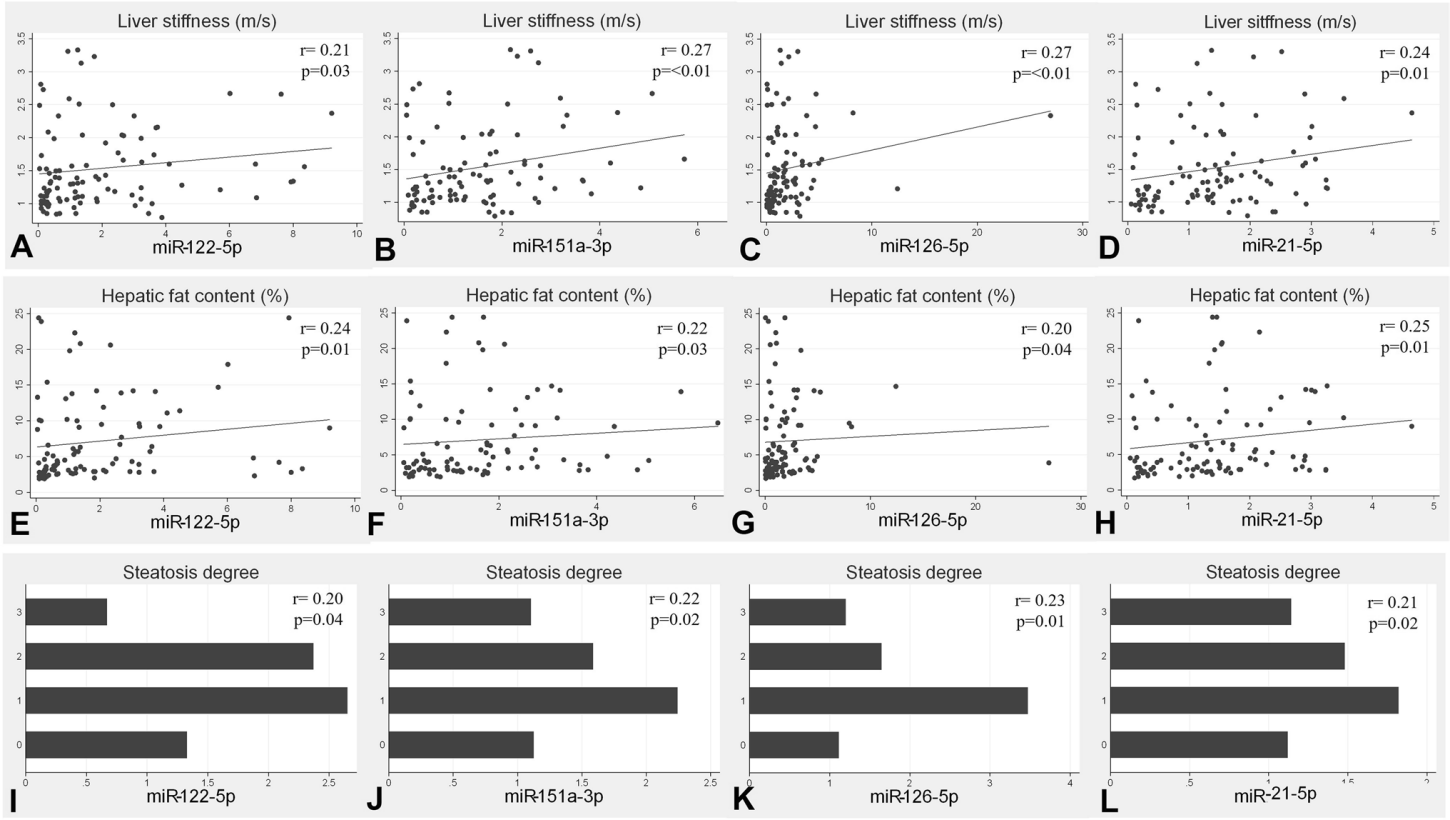
Correlation plots between liver stiffness, hepatic fat content, steatosis degree and miRNAs. Liver stiffness and (**A**) miR-122-5p, (**B**) miR-151a-3p, (**C**) miR-126-5p and (**D**) miR-21-5p. Hepatic fat content and (**E**) miR-122-5p, (**F**) miR-151a-3p, (**G**) miR-126-5p and (**H**) miR-21-5p. Steatosis degree and (**I**) miR-122-5p, (**J**) miR-151a-3p, (**K**) miR-126-5p and (**L**) miR-21-5p

Although they were not strong, positive associations were found between variables such as hepatic fat content, steatosis degree and liver stiffness and circulating miRNAs such as miR-122-5p, miR-151a-3p, miR-126-5p and miR-21-5p (Fig. 2).

Moreover, transaminases which are involved in MASLD, such as ALT (r = 0.46), AST (r = 0.38) and GGT (r = 0.47) were very positively correlated with miR-122-5p levels (p < 0.001) (Supplementary Tables 1, 2). Similarly, body composition and anthropometric measures were significantly associated with all miRNAs previously mentioned as well as HOMA index. Insulin was also positively associated with miR-122-5p, miR-151a-3p and miR-126-5p, and LECT2 was significantly correlated with miR-122-5p and miR-151a-3p. MiR-21-5p was also negatively associated with the MedDiet adherence score and adiponectin and was positively associated with chemerin.

### Logistic regression analyses for MASLD and related variables

Univariate analysis between hepatic fat content, steatosis degree and liver stiffness as dependent variables and miRNAs as independent variables (Tables 2, 3 and 4), demonstrated a moderate capacity to predict steatosis degree and liver stiffness (AUROCs 0.62–0.68). Furthermore, miR-21-5p was able to predict hepatic fat content with a moderate capacity (AUROC 0.62). MiR-126-5p was the only that did not predict liver stiffness (Tables 2, 3 and 4).

Contributing factors such as adiponectin, RBP4, TG, glucose, LECT2, leptin and chemerin were analyzed to evaluate its ability to predict MASLD-associated factors (Supplementary Table 3). The optimism-corrected values were used to validate AUROC results.

Multivariable regressions were performed including the contributing factors selected previously. The first model (model 1) was adjusted by sex, age and physical activity. The rest of the models were created from model 1 and adding different MASLD-related variables such as adiponectin (model 2), RBP4 (model 3), TG (model 4) and glucose (model 5), LECT (model 6), leptin (model 7) and chemerin (model 8).

**Fig. 3:**
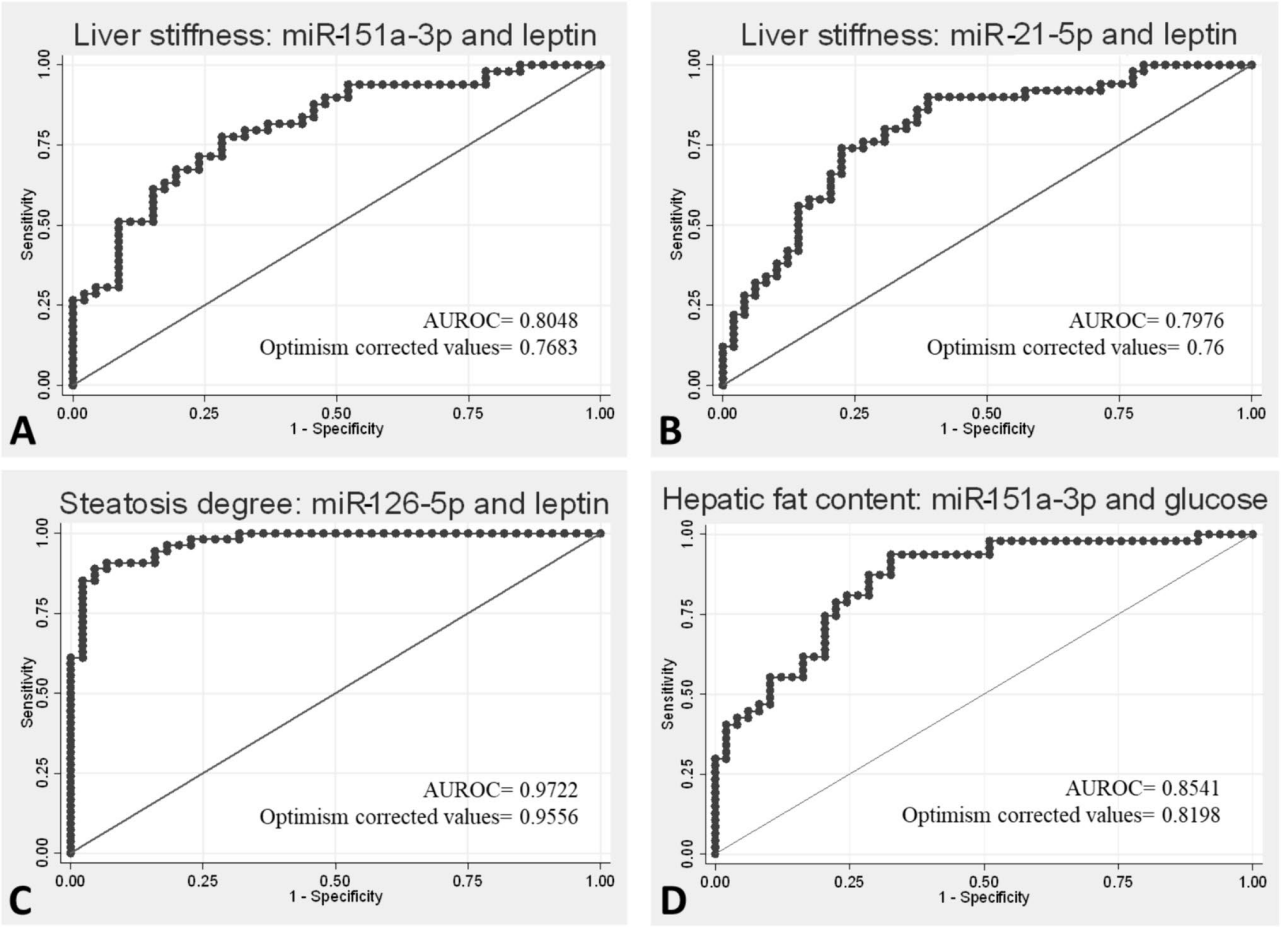
Receiver Operating Characteristic Curves for liver stiffness, hepatic steatosis degree and hepatic fat content with miRNAs. Multivariate models. Model 7: adjusted by sex, age, physical activity and leptin (**A**, **B**, **C**). Model 5: adjusted by sex, age, physical activity and glucose (**D**)

The most predictive capacity for liver stiffness with a moderate capacity was for miR-21-5p or miR-151a-3p both with leptin (model 7, AUC 0.76 in both, p < 0.05) (Table 2, Fig. 3). Other models with similar predictive capacity were miR-21-5p with chemerin, with an AUC of 0.70 and miR-151a-3p with LECT2 with an AUC of 0.72. Mir-122-5p with adiponectin had also predictive capacity but less that the other models with an AUC of 0.66. However, miR-126-5p was the only miRNA, that had not statistical significance in the predictive capacity for liver stiffness. For hepatic fat, miR-151a-3p with glucose (model 5) had the most ability to predict it with a high prediction capacity (model 5, AUC 0.81, p < 0.05, Fig. 3). In addition, miR-21-5p had also a high ability to predict hepatic fat with the third model that include RBP4 (AUC 0.80, p < 0.05,) (Table 3). Finally, the major value for predict the presence of steatosis was obtained combining miR-126-5p with leptin (model 7) obtaining an AUC of 0.95 (Fig. 3). A good capacity of prediction was achieved also using miR-122-5p, miR-151a-3p or miR-21-5p with adiponectin (model 2) with an AUC of 0.89 in all of them and miR-151-a3p or miR-21-5p with LECT2 (model 6) with an AUCs of 0.90, and 0.91, respectively (Table 4). We performed combinations of miRNAs with factors associated for MASLD (Supplementary Tables 4–6), but no combination was able to surpass the predictive capacity of each individual miRNAs.

**Table 2:** Logistic regressions analyses between liver stiffness as the dependent factors and miRNAs as predictive factors

	Liver stiffness (m/s)								
	Models	MiR-122-5p		MiR-151a-3p		MiR-126-5p		MiR-21-5p	
		P-value	AUROC	P-value	AUROC	P-value	AUROC	P-value	AUROC
Univariate		**0.028**	0.6321 (0.6246†)	** < 0.01**	0.6713 (0.6755†)	0.075	0.6620 (0.6585†)	** < 0.01**	0.6656 (0.6665†)
Multivariate (model 1 and others contributing variables)	**Model 1**	**0.034**	0.6808 (0.636†)	** < 0.01**	0.7186 (0.6755†)	0.130	0.6751 (0.6299†)	** < 0.01**	0.7260 (0.6859†)
	**Model 2**	**0.038**	0.7179 (0.6629†)	**0.011**	0.7286 (0.6832†)	0.189	0.7049 (0.6504†)	**0.014**	0.7372 (0.6866†)
	**Model 3**	**0.033**	0.6585 (0.6206†)	** < 0.01**	0.7260 (0.6692†)	0.131	0.6776 (0.6143)	** < 0.01**	0.7312 (0.6854†)
	**Model 4**	**0.038**	0.6971 (0.6418†)	** < 0.01**	0.7382 (0.6837†)	0.112	0.7049 (0.6497†)	** < 0.01**	0.7380 (0.6886†)
	**Model 5**	**0.033**	0.6813 (0,6183†)	** < 0.01**	0.7199 (0.6654†)	0.126	0.6788 (0.6141†)	** < 0.01**	0.7264 (0.6768)
	**Model 6**	0.066	0.7457 (0.7012†)	**0.011**	0.7695 (0.7215†)	0.161	0.7488 (0.7071†)	** < 0.01**	0.7488 (0.6972†)
	**Model 7**	0.075	0.8004 (0.7659†)	**0.017**	0.8048 (0.7683†)	0.201	0.7713 (0.7309†)	**0.010**	0.7976 (0.76†)
	**Model 8**	0.045	0.7233 (0.6739†)	**0.013**	0.7525 (0.6974†)	0.181	0.7167 (0.6632†)	**0.016**	0.7456 (0.7018†)

Abbreviations: AUROC, Area under the Receiver Operating Characteristic Curve; miR, microRNA; m/s, meters per second. † Optimism corrected AUROC value. P-value for the miRNAs in the logistic regression model. Models are adjusted. Model 1: adjusted by sex, age, physical activity. Model 2: adjusted by sex, age, physical activity and adiponectin. Model 3: adjusted by sex, age, physical activity and RBP4 (Retinol Binding Protein 4). Model 4: adjusted by sex, age, physical activity and triglycerides. Model 5: adjusted by sex, age, physical activity and glucose. Model 6: adjusted by sex, age, physical activity and LECT2 (Leukocyte cell-derived chemotaxin-2). Model 7: adjusted by sex, age, physical activity and leptin. Model 8: adjusted by sex, age, physical activity and chemerin

**Table 3:** Logistic regressions analyses between hepatic fat content as the dependent factors and miRNAs as predictive factors

	Hepatic fat content (%)								
	Models	MiR-122-5p		MiR-151a-3p		MiR-126-5p		MiR-21-5p	
		P-value	AUROC	P-value	AUROC	P-value	AUROC	P-value	AUROC
Univariate		0.064	0.6272 (0.615†)	0.053	0.6366 (0.6343†)	0.647	0.5955 (0.5689†)	**0.031**	0.6276 (0.6272†)
Multivariate (model 1 and others contributing variables)	**Model 1**	0.288	0.7580 (0.7236†)	0.127	0.7573 (0.7179†)	0.963	0.7510 (0.7095†)	0.096	0.7660 (0.7405†)
	**Model 2**	0.365	0.8405 (0.8097†)	0.216	0.8467 (0.8146†)	0.484	0.8392 (0.8102†)	0.312	0.8416 (0.8138†)
	**Model 3**	0.202	0.8322 (0.7885†)	0.052	0.8367 (0.7993†)	0.708	0.8159 (0.7773†)	**0.043**	0.8408 (0.8098†)
	**Model 4**	0.360	0.8180 (0.7776†)	0.061	0.8363 (0.7996†)	0.848	0.8171 (0.7744†)	0.097	0.8228 (0.7835†)
	**Model 5**	0.453	0.8421 (0.8116†)	**0.034**	0.8541 (0.8198†)	0.703	0.8404 (0.8071†)	0.080	0.8528 (0.8248†)
	**Model 6**	0.808	0.8491 (0.8246†)	0.477	0.8545 (0.819†)	0.913	0.8479 (0.8199†)	0.260	0.8612 (0.8391†)
	**Model 7**	0.437	0.8393 (0.806†)	0.209	0.8422 (0.8144†)	0.634	0.843 (0.817†)	0.118	0.8522 (0.834†)
	**Model 8**	0.372	0.7826 (0.7445†)	0.276	0.7772 (0.7341†)	0.736	0.7788 (0.7372†)	0.232	0.7892 (0.7553†)

Abbreviations: AUROC, Area under the Receiver Operating Characteristic Curve; miR, microRNA. † Optimism corrected AUROC value. P-value for the miRNAs in the logistic regression model. Models are adjusted. Model 1: adjusted by sex, age, physical activity. Model 2: adjusted by sex, age, physical activity and adiponectin. Model 3: adjusted by sex, age, physical activity and RBP4 (Retinol Binding Protein 4). Model 4: adjusted by sex, age, physical activity and triglycerides. Model 5: adjusted by sex, age, physical activity and glucose. Model 6: adjusted by sex, age, physical activity and LECT2 (Leukocyte cell-derived chemotaxin-2). Model 7: adjusted by sex, age, physical activity and leptin. Model 8: adjusted by sex, age, physical activity and chemerin

**Table 4:** Logistic regressions analyses between steatosis degree as the dependent factors and miRNAs as predictive factors

	Steatosis degree								
	Models	MiR-122-5p		MiR-151a-3p		MiR-126-5p		MiR-21-5p	
		P-value	AUROC	P-value	AUROC	P-value	AUROC	P-value	AUROC
Univariate		**0.016**	0.6591 (0.6571†)	** < 0.01**	0.6806 (0.6802†)	** < 0.01**	0.686 (0.6884†)	** < 0.01**	0.6626 (0.6598†)
Multivariate (model 1 and others contributing variables)	**Model 1**	**0.029**	0.7635 (0.7323†)	** < 0.01**	0.7661 (0.731†)	** < 0.01**	0.7675 (0.7348†)	**0.013**	0.7475 (0.7118†)
	**Model 2**	**0.036**	0.9116 (0.8938†)	**0.010**	0.9179 (0.892†)	**0.02**	0.9202 (0.8983†)	**0.107**	0.9083 (0.89†)
	**Model 3**	**0.012**	0.8443 (0.8133†)	** < 0.01**	0.8859 (0.8591†)	** < 0.001**	0.8815 (0.8603†)	** < 0.01**	0.8594 (0.8315†)
	**Model 4**	**0.039**	0.8283 (0.7925†)	** < 0.01**	0.8508 (0.8221†)	** < 0.01**	0.8646 (0.832†)	**0.013**	0.8307 (0.795†)
	**Model 5**	0.068	0.8476 (0.8162†)	** < 0.01**	0.8806 (0.8593†)	** < 0.01**	0.8815 (0.8551†)	**0.012**	0.8558 (0.8322†)
	**Model 6**	0.165	0.9177 (0.8937†)	**0.028**	0.9320 (0,9069†)	** < 0.01**	0.9497 (0.9293†)	**0.045**	0.9272 (0.9118†)
	**Model 7**	0.385	0.9668 (0.9521†)	0.091	0.9739 (0.9593†)	**0.049**	0.9722 (0.9556†)	0.217	0.9665 (0.9517†)
	**Model 8**	**0.040**	0.7883 (0.7471†)	**0.013**	0.7951 (0.7564†)	** < 0.001**	0.8815 (0.8588†)	**0.040**	0.7754 (0.7291†)

Abbreviations: AUROC, Area under the Receiver Operating Characteristic Curve; miR, microRNA. † Optimism corrected AUROC value. P-value for the miRNAs in the logistic regression model. Models are adjusted. Model 1: adjusted by sex, age, physical activity. Model 2: adjusted by sex, age, physical activity and adiponectin. Model 3: adjusted by sex, age, physical activity and RBP4 (Retinol Binding Protein 4). Model 4: adjusted by sex, age, physical activity and triglycerides. Model 5: adjusted by sex, age, physical activity and glucose. Model 6: adjusted by sex, age, physical activity and LECT2 (Leukocyte cell-derived chemotaxin-2). Model 7: adjusted by sex, age, physical activity and leptin. Model 8: adjusted by sex, age, physical activity and chemerin

### *In silico *evaluation

The *in silico* analysis to further explore the potential role of miRNA in MASLD revealed that several genes associated with MASLD are regulated by specific microRNAs (Supplementary Table 7). Genes such as *CREB1, MAPK1* or *PTPN2* are extensively linked to MASLD and regulated by miR-122-5p. Additionally, miR-126-5p regulates important genes like *SORT1* and *PRKAA2*, while miR-151a-3p regulates *HADH* and *MCL1*, as well as others. Furthermore, miR-21-5p regulates genes such as *CCR7* and *DNM1L*, including additional ones. All these genes are regulated through different signaling pathways including JAK-STAT, PI3K-Akt, TNFα, chemerin, NOD-like receptor, mTOR, among others (Supplementary Tables 8–11). Additionally, the top 10 metabolic pathways according to p-adjusted value were depicted for the target genes associated with each miRNA in the Supplementary Fig. 2. Furthermore, a network of interactions between our specific miRNAs and their target genes were generated. The network shows a high density of interactions, with 4042 target genes identified. Some genes, such as *CALD1, CCNG1, TNPO1, PURB, MYCBP2, MTPN, SNTB2, CBX5, CREBRF, BRWD1* and *RBM12* were shared by miR-122-5p, miR-151-3p, miR-21-5p and miR-126-5p (Supplementary Fig. 3).

## Discussion

MASLD is becoming a global health challenge, and there is a need for newer disease prediction and prognosis biomarker. Liver biopsy is the reference technique for diagnosis and prognosis of MASLD, however it has limitations and carries a risk for the patient because it is an invasive and complex method [30], so in this sense, the need arises to continue investigating new methods allowing to know the stage of the disease in a simple and more informative way.

Non-invasive imaging techniques like ultrasound, CT, MRI, and proton magnetic spectroscopy aim to replace biopsies but depend on operator skills and costly equipment. Ultrasound lacks objective analysis, while quantitative methods like proton density fat fraction (PDFF) require specialized equipment. Transient elastography (TE), the most common non-invasive MASLD diagnostic tool, has significant sampling variability, with probe positioning affecting results in over 30% of patients [58]. ARFI can have diagnostic accuracy comparable to that of TE, indicating its potential as a valuable tool in liver assessment. However, ARFI has limitations, including the requirement for the operator to define the region of interest and obtain a series of liver stiffness measurements (LSM) [33].

Despite the intricate biological complexity of miRNAs, they serve as reliable circulating biomarkers for MASLD diagnosis across various disease stages [39]. These small non-coding RNA molecules, typically ≤ 25 nucleotides long, regulate gene expression post-transcriptionally by binding to the 3' untranslated region of their target mRNAs [43]. MASLD is associated with alterations in hepatic miRNA expression patterns across early, intermediate, and advanced stages. Specific miRNA species are implicated in steatosis development and the progression of MASL to MASH and cirrhosis [30]. MiRNAs are excellent biomarkers due to their high stability, protected by vesicles and proteins, enabling resistance to external insults. Detection techniques based on PCR are extremely sensitive, capable of identifying even a single molecule [17]. Additionally, miRNAs can be detected in various bodily fluids including serum, plasma, whole blood, urine, and saliva. These miRNAs remain stable under different conditions such as temperature, pH variations, and over time, making them easy to measure, making them useful as diagnostic and prognostic indicator for diseases [11].

In the present study, we were able to observe that those participants with MASLD, whom had hepatic steatosis proven by ultrasonography, clearly had higher fat content and liver stiffness than in the control group, whom had lack of the disease. Elevated values of liver transaminases such as ALT and GGT, but not AST, were observed in MASLD group in comparison with control group. Moreover, it had been shown that ALT and GGT are biomarkers of liver disease, however AST could be more related with alcoholic liver or autoimmune diseases [53].

MiRNAs are interrelated to both inflammation and metabolic control [38]. HOMA-IR, HbA1c, insulin and blood glucose were increased in MASLD group in comparison with control group. HOMA-IR had been showed to be a good predictor for hepatic steatosis and fat content and insulin as a risk factor for MASLD [18]. As well, in hepatic IR, insulin, continues stimulating lipogenesis, which ends up producing hyperglycemia, hyperlipidemia, hepatic steatosis and T2DM [10]. MASLD can be associated with disturbances in glucose metabolism, and subsequently, elevated blood glucose levels may support the fat accumulation. In hepatic steatosis, the hepatic glucose utilization rate is increased, possibly as a result of IR and elevated insulin levels in the bloodstream [15, 34]. Despite healthier lifestyles, the control group showed higher LDL-c and TC levels, not yet reaching pathological thresholds [27]. Elevated HDL-c in controls might drive the TC increase, while low HDL-c links to MASLD [16]. MASLD group exhibited significantly increased TG levels, crucial in MASLD pathogenesis by promoting fatty acid deposition, aligning with previous studies considering TG as a risk factor for MASLD [65]. Additionally, higher atherogenic indices in MASLD suggest elevated cardiovascular risk and less favorable lipid profiles compared to controls [23].

A decreasing in adiponectin levels was observed in the MASLD group. Adiponectin acts as a protective agent against hepatic steatosis, inflammation, and fibrosis [24]. Similarly, we found adiponectin to be a protective factor against MASLD, predicting hepatic fat content, steatosis degree, and liver stiffness. Additionally, LECT2, leptin, and chemerin levels were increased in subjects with MASLD. Previous observations indicate a general increase in chemerin and LECT2 levels in obesity and IR states [36, 49]. Leptin had been observed to increase with the severity of MASLD, suggesting a possible compensatory mechanism against fat accumulation [49]. Recent studies showed that RBP4 levels in adipose and circulating tissue are associated with IR, dyslipidemia and T2DM and therefore linked to MASLD [47]. Our results showed that RBP4, in addition to being elevated in subjects with MASLD compared to the control group, could predict hepatic steatosis and hepatic fat content with a moderate capacity.

Furthermore, our miRNAs analyses revealed that only miR-21-5p, miR-151a-3p, miR-126-5p and miR-122-5p had significantly higher circulating levels in patients from the MASLD group compared with controls. Previous studies demonstrated that miR-122-5p was increased and positively correlated with markers of MASLD severity [66] and with body weight, TG, and body insulin insensitivity [2] and distinguished MASLD from healthy controls [39]. Similar, higher levels of miR-21 are effective biomarkers for MASLD diagnosis and play a key role in the development of the disease [54]. Other study showed that circulating miR-15b-3p, miR-21-5p, miR-29b-3p, miR-126-5p, miR-151a-3p and miR-192-5p were increased more than twice in a NASH group compared with the MASLD group [35], indicating a relation between miRNAs and the severity of the disease.

The analysis focused on miRNAs significantly differing between groups, finding positive associations between body composition, anthropometric measures, and all miRNAs. The MASLD group had lower adherence to the MedDiet and higher energy intake, aligning with obesity being a key factor in MASLD development, consistent with the negative correlation between miRNAs and MedDiet observed in our study. It had been shown that a balanced nutrition and moderate weight loss were the best therapeutic approach for MASLD [3]. Additionally, significant negative correlations were observed between our four miRNAs and adiponectin, particularly evident in miR-21-5p, supporting the documented role of miR21 in upregulating adiponectin mRNA expression [32]. On the other hand, chemerin, which participates in regulating angiogenesis, inflammation and cell proliferation [49], was positively correlated with miR-21-5p, which is positively correlated with steatosis, lobular inflammation, serum ALT and hepatic activity [66]. LECT2 positively correlated with miR-122-5p and miR-151a-3p, paralleling its significant levels in MASLD individuals with metabolic syndrome components and associations with obesity and anthropometric measures [36]. Likewise, liver stiffness, steatosis, and hepatic fat content correlated with miR-21-5p, miR-122-5p, miR-151a-3p, and miR-126-5p, consistent with findings in other studies [7, 20, 50, 64].

Our logistic regression analyses demonstrated the predictive capability of miRNAs for MASLD, even after adjusting for potential variables related to the disease such as leptin or glucose, among others. We obtained that the combination of miR-21-5p or miR-151a-3p with leptin for predict liver stiffness with an AUC of 0.76 for both options, miR-151a-3p with glucose was also the best combination to predict the hepatic fat content with an AUC of 0.81, and miR-126-5p with leptin to predict the presence or absence of hepatic steatosis and therefore, the disease, with an AUC of 0.95. Similar results were obtained in other study in which miR-122 discriminate subjects with MASLD from controls with an AUC of 0.85 [52]. In addition, it had been shown that miR-21-5p, miR151a-3p and miR-126,5p individually showed a diagnostic accuracy for MASH with an AUC of 0.73, 0.75 and 0.69, respectively for MASH as well as the combination of miR-21-5p, miR-151a-3p, miR-192-5p and miR-4449 with an AUC of 0.87 [35]. Furthermore, other study included miR-122-5p, miR-1290, miR-27b-3p and miR-192-5p with An AUC of 0.85 for MASLD [56]. In our study the combination of various miRNAs together does not provide better results than the miRNA individually for the diagnosis of our variables associated with MASLD suggesting that each miRNA could had been involved differently depending on the stage of the disease and could impact other miRNAs by predicting MASLD. Other non-invasive scores such as FLI index, Hepatic Steatosis Index (HIS), Steatotest, and NAFLD Liver fat Score showed a moderate diagnostic performance for MASLD (AUC from 0.68- 0.87), but it is important to highlight that the integration of these scores into everyday clinical practice is often limited by concerns about their diagnostic effectiveness [1]. Our study provides evidence of the predictive capacity of miR-122-5p, miR-151a-3p, miR-126-5p and miR-21-5p for hepatic steatosis, hepatic stiffness, and fat content, offering a non-invasive and promising method compared to liver biopsy and others non-invasive tools with a higher diagnostic performance. Furthermore, it integrates multiple diagnostic modalities and clinical variables for a comprehensive disease evaluation, promoting a personalized approach in medicine.

When we performed the *in silico* analysis, we found different target genes involved in MASLD, which are being regulated by the four miRNAs and which participate and contribute to the development and progression of the disease. In general, these genes are implicated in the regulation of lipid metabolism, inflammatory response, energy homeostasis and apoptosis in the liver through numerous pathways such as PI3K-AKT, JAK-STAT, mTOR, AMPK, cAMP, NF-kappa B, IL-6 and TNFα signaling pathways, among others [13, 19]. In addition, identifying shared target genes is also important as it underscores the complexity of gene regulation by multiple miRNAs. Such interactions may have significant implications for biological processes and disease mechanisms, underscoring the importance of considering miRNA co-regulation in molecular studies. Their dysfunctions can contribute to the accumulation of hepatic fat, chronic inflammation and metabolic imbalance observed in the development and progression of MASLD. Advancing our knowledge about this molecular mechanism will provide a better understanding of the diseases and enable the development of more effective therapeutic approaches. Studying the expression of miRNAs of interest in MASLD is crucial to understand its pathogenesis, diagnose the condition, and identify potential therapeutic targets. Additionally, their expression in various tissues can help in the development of miRNA-based therapies and serve as biomarkers for disease assessment.

However, the study has some limitations that should be acknowledged. First, miRNA level expression was normalized with the exogenous Unisp6, suggested by Vigneron et al. [59]. Not all studies had been controlled with this reference gene. Further evaluations are required using other different standardized controls in the applications of miRNAs as potential biomarkers for MASLD for reducing the technical variability among experimental replicates. Secondly, for the evaluation of MASLD non-invasive imaging techniques for the assessment of hepatic steatosis were used instead of a liver biopsy, which is the most reliable method in the detection of MASH or fibrosis in MASLD patients. However, this method is an invasive procedure, very expensive, with possible error in samples and complications that occurs during it procedure [66]. Thirdly, in our study, MASLD group consisted of individuals with MASLD and obesity, while our controls were subjects with normal-weight without MASLD. Although it would have been more interesting to have controls without MASLD and with obesity, it was challenging because most of the patients with obesity that we recruited, had MASLD. Ensuring uniform sample characteristics in a scientific study is crucial for maintaining research quality, result validity, and facilitating other researchers; ability to comprehend, replicate, and build upon your work, thus advancing scientific knowledge effectively and reliably. Fifthly, we only evaluated a few specific miRNAs. A comprehensive analysis of the full miRNA profiles in the blood could have identified other miRNAs that, alone or in combination, might serve as better predictors. Lastly, it is worth noting that this study evaluated a sample of patients at baseline. MicroRNAs (miRNAs), as epigenetic modifiers, can determine not only early disease evaluation but also the risk of progression and prognosis. Therefore, it would be highly interesting to observe changes in miRNA expression over time and assess differential aspects in miRNA expression patterns when different diets are applied.

## Conclusion

In summary, findings of this study demonstrated that the combination of miRNAs including miR-151a-3p or miR-21-5p or miR-126-5p along leptin and miR-151a-3p with glucose can be used as non-invasive biomarker for the comprehensive assessment of steatosis, liver stiffness and hepatic fat content, which are critical factors in determining the presence of MASLD. These findings do not only highlight the promise of miRNAs as epigenetic regulators but also accentuate their application in the early assessment of the disease with potential in precision medicine.

## Supplementary Information

Below is the link to the electronic supplementary material.

Supplementary file1 (DOCX 1163 KB)

## Data Availability

Data available on request due to privacy/ethical restrictions by sending an email to the corresponding author.
