# Supplementary material for: Circulating miR-122-5p, miR-151a-3p, miR-126-5p and miR-21-5p as potential predictive biomarkers for Metabolic Dysfunction-Associated Steatotic Liver Disease assessment
Source: J Physiol Biochem. 2024 Aug 14;81(4):1275–88. doi: 10.1007/s13105-024-01037-8 (PMC12738678; doi:10.1007/s13105-024-01037-8)
Supplement: Supplementary file 1 — Supplementary file1 (DOCX 1163 KB) [file 13105_2024_1037_MOESM1_ESM.docx]

**Circulating miR-122-5p, miR-151a-3p, miR-126-5p and miR-21-5p as potential predictive biomarkers for Metabolic Dysfunction-Associated Steatotic Liver Disease assessment**

*J Physiol Biochem*

Ana Luz Tobaruela-Resola ^1^, Fermín I. Milagro ^1,2,3^, Mariana Elorz ^2,4,^ Alberto Benito-Boillos^2,4^, José I. Herrero ^2,5,6^, Paola Mogna-Peláez ^1^, Josep A. Tur ^3,7^, J. Alfredo Martínez ^3,8^, Itziar Abete ^1,2,3#^, M. Ángeles Zulet ^1,2,3#*^

^1^Department of Nutrition, Food Sciences and Physiology and Centre for Nutrition Research, Faculty of Pharmacy and Nutrition, Centre for Nutrition Research, University of Navarra, 31008 Pamplona, Spain.

^2^Navarra Institute for Health Research (IdiSNA), 31008 Pamplona, Spain.

^3^Biomedical Research Centre Network in Physiopathology of Obesity and Nutrition (CIBERobn), Instituto de Salud Carlos III, 28029 Madrid, Spain.

^4^Department of Radiology, Clínica Universidad de Navarra, 31008 Pamplona, Spain.

^5^Liver Unit, Clínica Universidad de Navarra, 31008 Pamplona, Spain.

^6^Biomedical Research Centre Network in Hepatic and Digestive Diseases (CIBERehd), 28029 Madrid, Spain

^7^Research group on Community Nutrition and Oxidative Stress, University of Balearic Islands, 07122 Palma, Spain.

^8^Precision Nutrition and Cardiovascular Health Program, IMDEA Food, CEI UAM + CSIC, Madrid Spain.

#These authors contributed equally to this work.

*Authors to whom correspondence should be addressed.

[mazulet@unav.es](mailto:mazulet@unav.es).

**
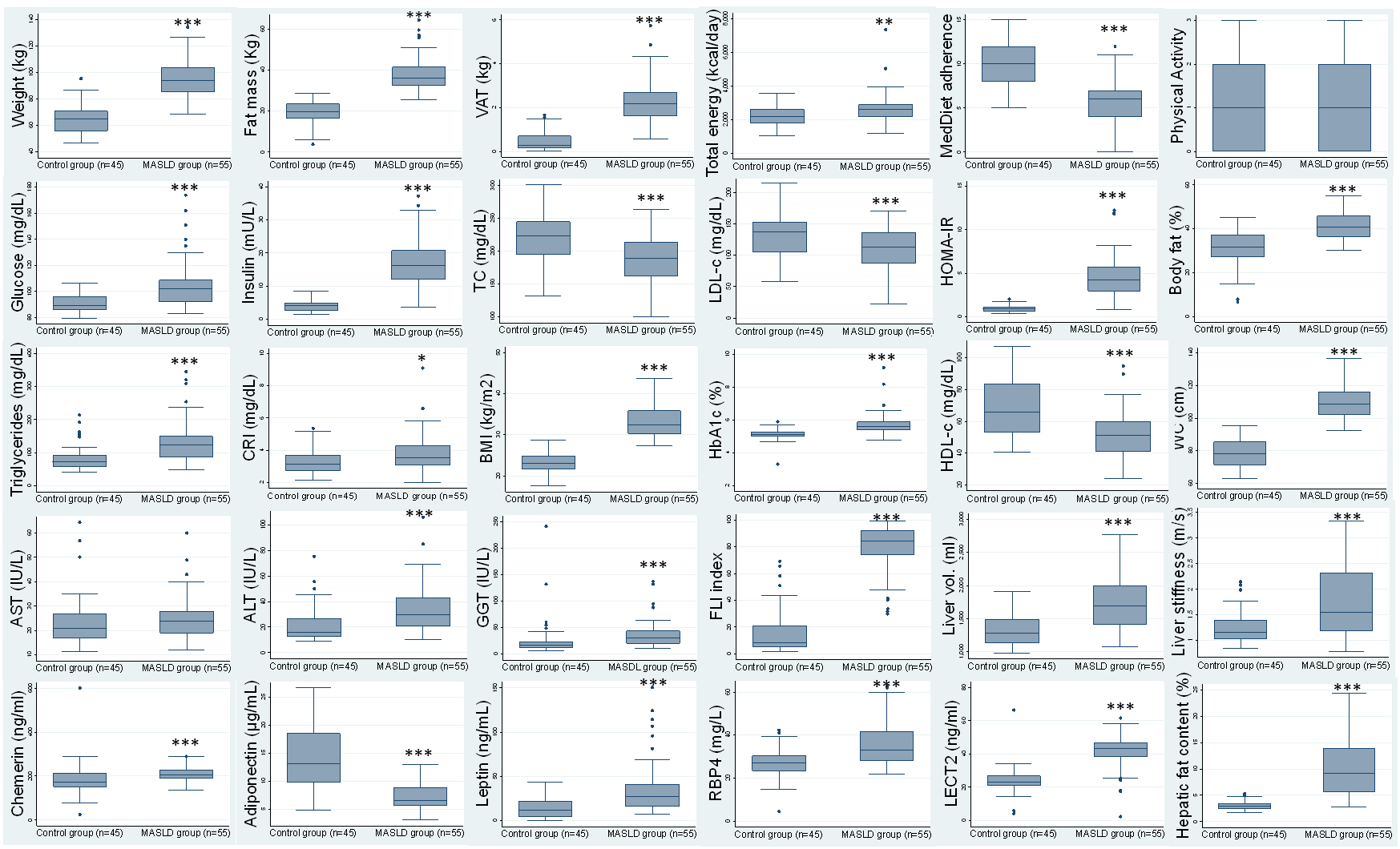
**

**Supplementary Figure 1.** Box and whisker plots of body composition, biochemical determinations, lifestyle parameters, inflammatory markers and hepatic status of the Control and MASLD groups of the study are shown. P-value: p<0.05*; p<0.01**; p<0.001***. Abbreviations: BMI, Body Mass Index; VAT, Visceral Adipose Tissue; MedDiet, Mediterranean Diet; FLI, Fatty Liver Index; ALT, Alanine aminotransferase; AST, Aspartate aminotransferase; GGT, Gamma-glutamyl transferase; HbA1C, Hemoglobin A1C; HOMA-IR, Homeostatic model Assessment for Insulin Resistance; TC, Total Cholesterol; HDL-c, High Density Lipoprotein cholesterol; LDL-c, Low Density Lipoprotein cholesterol; CRI, Castelli Risk Index; LECT2, Leukocyte cell-derived chemotaxin-2; RBP4, Retinol Binding Protein.

**Supplementary Table 1**. Correlations between miR-122-5p, miR-151a-3p, miR-126-5p, miR-21-5p and body composition, anthropometrics, lifestyle parameters and hepatic status.

| **MASLD and Control group (n=100)** | **MiR-122-5p** | | **MiR-151a-3p** | | **MiR-126-5p** | | **MiR-21-5p** | |
| --- | --- | --- | --- | --- | --- | --- | --- | --- |
|  | **Correl. Coef.** | **P-value** | **Correl. Coef.** | **P-value** | **Correl. Coef.** | **P-value** | **Correl. Coef.** | **P-value** |
| **Body composition, lifestyle markers and hepatic status** | | | | | | | | |
| Weight (kg) | 0.2943 | **<0.01** | 0.2677 | **<0.01** | 0.2628 | **0.008** | 0.2919 | **<0.01** |
| BMI (kg/m2) | 0.2486 | **0.013** | 0.2016 | **0.048** | 0.2241 | **0.025** | 0.2382 | **0.017** |
| VAT (kg) | 0.3033 | **<0.01** | 0.2424 | **0.017** | 0.2479 | **0.013** | 0.2497 | **0.012** |
| Body fat (%) | 0.0475 | 0.644 | 0.1375 | 0.184 | 0.1244 | 0.222 | 0.0816 | 0.421 |
| Waist circumference (cm) | 0.2780 | **<0.01** | 0.2648 | **0.021** | 0.2499 | **0.012** | 0.2537 | **0.010** |
| Fat mass (Kg) | 0.2179 | **0.032** | 0.2631 | **0.010** | 0.2579 | **0.010** | 0.2265 | **0.024** |
| MedDiet adherence score | -0.2334 | **0.020** | -0.1536 | 0.135 | -0.1186 | 0.242 | -0.1967 | **0.049** |
| Total energy (kcal/day) | 0.2432 | **0.018** | 0.1052 | 0.318 | 0.1395 | 0.177 | 0.1120 | 0.277 |
| Physical Activity | -0.1504 | 0.287 | 0.0571 | 0.690 | -0.1290 | 0.362 | 0.0101 | 0.943 |
| Steatosis degree | 0.2020 | **0.046** | 0.2287 | **0.025** | 0.2368 | **0.018** | 0.2187 | **0.028** |
| Hepatic fat content (%) | 0.2498 | **0.014** | 0.2226 | **0.032** | 0.2063 | **0.043** | 0.2537 | **0.012** |
| liver vol. (ml) | 0.0704 | 0.499 | 0.0496 | 0.638 | 0.0547 | 0.600 | 0.1078 | 0.298 |
| Liver stiffness (m/s) | 0.2165 | **0.033** | 0.2481 | **0.015** | 0.2575 | **0.010** | 0.2355 | **0.018** |
| FLI index | 0.2978 | **<0.01** | 0.1721 | 0.093 | 0.2004 | **0.046** | 0.2400 | **0.016** |
| ALT (IU/L) | 0.4627 | **<0.001** | 0.1956 | 0.056 | 0.1761 | 0.081 | 0.2794 | **<0.01** |
| AST (IU/L) | 0.3885 | **<0.001** | 0.0948 | 0.358 | 0.0965 | 0.341 | 0.1938 | 0.053 |
| GGT (IU/L) | 0.4774 | **<0.001** | 0.1542 | 0.133 | 0.1631 | 0.106 | 0.2532 | **0.011** |

Abbreviations: MASLD, Metabolic Dysfunction-Associated Steatotic Liver Disease; BMI, Body Mass Index; VAT, Visceral Adipose Tissue; ; Med Diet score, Mediterranean Diet Adherence Score; FLI, Fatty Liver Index; ALT, Alanine aminotransferase; AST, Aspartate aminotransferase; GGT, Gamma-glutamyl transferase.

**Supplementary Table 2**. Correlations between miR-122-5p, miR-151a-3p, miR-126-5p, miR-21-5p and biochemical determinations and inflammatory markers.

| **MASLD and Control group (n=100)** | **MiR-122-5p** | | **MiR-151a-3p** | | **MiR-126-5p** | | **MiR-21-5p** | |
| --- | --- | --- | --- | --- | --- | --- | --- | --- |
|  | **Correl. Coef.** | **P-value** | **Correl. Coef.** | **P-value** | **Correl. Coef.** | **P-value** | **Correl. Coef.** | **P-value** |
| **Biochemical determinations and inflammatory markers** | | | | | | | | |
| Glucose (mg/dL) | 0.1873 | 0.064 | 0.0164 | 0.874 | 0.0513 | 0.614 | 0.0656 | 0.516 |
| Insulin (mU/L) | 0.3101 | **<0.01** | 0.2851 | **<0.01** | 0.2287 | 0.022 | 0.1888 | 0.060 |
| HbA1c (%) | 0.1581 | 0.120 | 0.1552 | 0.131 | 0.0781 | 0.442 | 0.1671 | 0.096 |
| HOMA-IR | 0.2871 | **<0.01** | 0.2478 | **0.014** | 0.2084 | **0.038** | 0.2323 | **0.020** |
| TC (mg/dL) | -0.0909 | 0.373 | -0.1226 | 0.233 | -0.1673 | 0.097 | -0.0701 | 0.488 |
| HDL-c (mg/dL) | -0.1690 | 0.096 | -0.0931 | 0.366 | -0.0761 | 0.454 | -0.1077 | 0.286 |
| LDL-c (mg/dL) | -0.0474 | 0.642 | -0.0601 | 0.560 | -0.1272 | 0.209 | -0.0542 | 0.591 |
| CRI (mg/dL) | 0.1627 | 0.109 | 0.0695 | 0.501 | 0.0089 | 0.930 | 0.0884 | 0.381 |
| Triglycerides (mg/dL) | 0.1459 | 0.151 | -0.0531 | 0.607 | -0.0158 | 0.876 | 0.0856 | 0.397 |
| Chemerin (ng/ml) | 0.1122 | 0.271 | 0.1195 | 0.246 | 0.0729 | 0.473 | 0.2451 | **0.014** |
| LECT2 (ng/ml) | 0.2663 | **<0.01** | 0.2189 | **0.033** | 0.1193 | 0.242 | 0.1968 | 0.050 |
| RBP4 (mg/l) | 0.0947 | 0.353 | -0.0574 | 0.578 | -0.0699 | 0.491 | 0.0139 | 0.890 |
| Leptin (ng/mL) | 0.0634 | 0.537 | 0.1866 | 0.070 | 0.1207 | 0.236 | 0.1152 | 0.256 |
| Adiponectin (μg/mL) | -0.1498 | 0.140 | -0.1053 | 0.307 | -0.1523 | 0.132 | -0.2486 | **0.012** |

Abbreviations: MASLD, Metabolic Dysfunction-Associated Steatotic Liver Disease; HbA1C, Hemoglobin A1C; HOMA-IR, Homeostatic model Assessment for Insulin Resistance; TC, Total Cholesterol; HDL-c, High Density Lipoprotein cholesterol; LDL-c, Low Density Lipoprotein cholesterol; CRI, Castelli Risk Index; LECT2, Leukocyte cell-derived chemotaxin-2; RBP4, Retinol Binding Protein 4.

**Supplementary Table 3.** Logistic regression analyses between liver stiffness, steatosis degree and hepatic fat content as the dependent factors and adiponectin, RBP4, triglycerides (TG), glucose, LECT2, leptin and chemerin as predictive factors.

| **Models** | **Liver stiffness (m/s)** | | **Steatosis degree** | | **Hepatic fat content (%)** | |
| --- | --- | --- | --- | --- | --- | --- |
|  | **Univariate models** | | | | | |
|  | **P-value** | **AUROC** | **P-value** | **AUROC** | **P-value** | **AUROC** |
| **Adiponectin** | **0.037** | 0.5876 (0.5885ⴕ) | **<0.001** | 0.8554 (0.8574ⴕ) | **<0.001** | 7762 (0.7767ⴕ) |
| **RBP4** | 0.399 | 0.6124 (0.577ⴕ) | **<0.001** | 0.7667 (0.7654ⴕ) | **<0.001** | 0.7638 (0.7635ⴕ) |
| **TG** | 0.094 | 0.6254 (0.6175ⴕ) | **<0.001** | 0.7776 (0.7775ⴕ) | **<0.001** | 0.7838 (0.786ⴕ) |
| **Glucose** | 0.192 | 0.6222 (0.6133ⴕ) | **<0.001** | 0.8024 (0.797ⴕ) | **<0.001** | 0.7958 (0.7931ⴕ) |
| **LECT2** | **<0.01** | 0,6753 (0,6725ⴕ) | **<0.001** | 0.9045 (0.9063ⴕ) | **<0.001** | 0.8018 (0.8021ⴕ) |
| **Leptin** | **0.017** | 0.6476 (0.6511ⴕ) | **<0.001** | 0.7948 (0.7925ⴕ) | **0.041** | 0.6682 (0.6613ⴕ) |
| **Chemerin** | 0.069 | 0.6692 (0.6763ⴕ) | **0.05** | 0.7006 (0.6967ⴕ) | 0.081 | 0.6858 (0.6827ⴕ) |

Abbreviations: AUROC: Area under the ROC curves; m/s, meters per second; TG, Triglycerides; RBP4, Retinol Binding Protein 4; LECT2, Leukocyte cell-derived chemotaxin-2.

**Supplementary Table 4.** Logistic regressions analyses between liver stiffness and miRNAs combinations.

| **Models** | | **Liver stifness (m/s)** | | | | | |
| --- | --- | --- | --- | --- | --- | --- | --- |
|  |  | **MiR-151a-3p + MiR-122-5p** | | **MiR-151a-3p + MiR-126-5p** | | **MiR-21-5p + MiR-126-5p** | |
|  |  | **P-value** | **AUROC** | **P-value** | **AUROC** | **P-value** | **AUROC** |
| **Univariate** | | **0.035** | 0.6809 (0.6709ⴕ) | **0.012** | 0.6882 (0.6732ⴕ) | **0.012** | 0.6939 (0.6775ⴕ) |
| **Multivariate (model 1 and others contributing variables)** | **Model 1** | **0.045** | 0.7264 (0.6698ⴕ) | **0.014** | 0.7317 (0.6769ⴕ) | **0.017** | 0.7367 (0.6842ⴕ) |
|  | **Model 2** | 0.064 | 0.7347 (0.6744ⴕ) | **0.019** | 0.7338 (0.6773ⴕ) | **0.027** | 0.7424 (0.6876ⴕ) |
|  | **Model 3** | **0.037** | 0.7243 (0.6658ⴕ) | **0.013** | 0.7338 (0.6736ⴕ) | **0.017** | 0.7367 (0.6763ⴕ) |
|  | **Model 4** | **0.024** | 0.7360 (0.6839ⴕ) | **<0.01** | 0.7434 (0.6883ⴕ) | **0.016** | 0.7461 (0.6889ⴕ) |
|  | **Model 5** | **0.037** | 0.7230 (0.663ⴕ) | **0.012** | 0.7312 (0.6678ⴕ) | **0.016** | 0.7367 (0.6755ⴕ) |
|  | **Model 6** | **0.044** | 0.7673 (0.7145ⴕ) | **0.022** | 0.7793 (0.7226ⴕ) | **0.020** | 0.7833 (0.7313ⴕ) |
|  | **Model 7** | 0.067 | 0.8118 (0.7679ⴕ) | **0.022** | 0.8052 (0.7597ⴕ) | **0.017** | 0.8009 (0.7548ⴕ) |
|  | **Model 8** | 0.072 | 0.7577 (0.6988ⴕ) | **0.024** | 0.756 (0.699ⴕ) | **0.032** | 0.7576 (0.7021ⴕ) |
| **Models** | | **MiR-21-5p + MiR-151a-3p** | | **MiR-122-5p + MiR-126-5p** | | **MiR-21-5p + MiR-122-5p** | |
|  |  | **P-value** | **AUROC** | **P-value** | **AUROC** | **P-value** | **AUROC** |
| **Univariate** | | 0.701 | 0.6843 (0.6643ⴕ) | 0.070 | 0.6679 (0.6533ⴕ) | 0.085 | 0.6617 (0.6403ⴕ) |
| **Multivariate (model 1 and others contributing variables)** | **Model 1** | 0.804 | 0.7212 (0.6631ⴕ) | 0.072 | 0.6977 (0.6387ⴕ) | 0.093 | 0.7196 (0.6652ⴕ) |
|  | **Model 2** | 0.930 | 0.7317 (0.6653ⴕ) | 0.066 | 0.7249 (0.6638ⴕ) | 0.176 | 0.7417 (0.6822ⴕ) |
|  | **Model 3** | 0.821 | 0.7256 (0.6556ⴕ) | 0.072 | 0.6977 (0.6298ⴕ) | 0.090 | 0.7204 (0.6597ⴕ) |
|  | **Model 4** | 0.900 | 0.7382 (0.6802ⴕ) | 0.087 | 0.7151 (0.656ⴕ) | 0.075 | 0.7304 (0.6691ⴕ) |
|  | **Model 5** | 0.812 | 0.7225 (0.6525ⴕ) | 0.072 | 0.7028 (0.6349ⴕ) | 0.089 | 0.7204 (0.6598ⴕ) |
|  | **Model 6** | 0.681 | 0.7691 (0.7087ⴕ) | 0.118 | 0.76 (0.7042ⴕ) | 0.087 | 0.7662 (0.7126ⴕ) |
|  | **Model 7** | 0.985 | 0.8048 (0.7608ⴕ) | 0.106 | 0.7968 (0.7522ⴕ) | 0.171 | 0.8085 (0.767ⴕ) |
|  | **Model 8** | 0.916 | 0.7512 (0.6887ⴕ) | 0.079 | 0.7317 (0.6711ⴕ) | 0.187 | 0.745 (0.6874ⴕ) |

Abbreviations: AUROC, Area under the Receiver Operating Characteristic Curve; miR, microRNA; m/s, meters per second. ⴕ Optimism corrected AUROC value. P-value for the miRNAs in the logistic regression model. Models are adjusted. Model 1: adjusted by sex, age, physical activity. Model 2: adjusted by sex, age, physical activity and adiponectin. Model 3: adjusted by sex, age, physical activity and RBP4 (Retinol Binding Protein 4). Model 4: adjusted by sex, age, physical activity and triglycerides. Model 5: adjusted by sex, age, physical activity and glucose. Model 6: adjusted by sex, age, physical activity and LECT2 (Leukocyte cell-derived chemotaxin-2). Model 7: adjusted by sex, age, physical activity and leptin. Model 8: adjusted by sex, age, physical activity and chemerin.

**Supplementary Table 5**. Logistic regressions analyses between steatosis degree and combinations of miRNAs.

| **Models** | | **Steatosis degree** | | | | | |
| --- | --- | --- | --- | --- | --- | --- | --- |
|  |  | **MiR-151a-3p + MiR-122-5p** | | **MiR-151a-3p + MiR-126-5p** | | **MiR-21-5p + miR-126-5p** | |
|  |  | **P-value** | **AUROC** | **P-value** | **AUROC** | **P-value** | **AUROC** |
| **Univariate** | | **0.037** | 0.6955 (0.6701ⴕ) | 0.392 | 0.6994 (0.6822ⴕ) | 0.603 | 0.6959 (0.6766ⴕ) |
| **Multivariate (model 1 and others contributing variables)** | **Model 1** | **0.032** | 0,7657 (0.7189ⴕ) | 0.245 | 0.7802 (0.7419ⴕ) | 0.724 | 0.7663 (0.7264ⴕ) |
|  | **Model 2** | 0.052 | 0.9021 (0.8979ⴕ) | **0.045** | 0.9272 (0.9067ⴕ) | 0.79 | 0.9214 (0.8967ⴕ) |
|  | **Model 3** | **<0.01** | 0.8885 (0.8548ⴕ) | 0.309 | 0.8956 (0.8613ⴕ) | **0.012** | 0.8815 (0.8457ⴕ) |
|  | **Model 4** | **<0.01** | 0.8534 (0.8153ⴕ) | 0.124 | 0.8684 (0.8285ⴕ) | **0.02** | 0.8663 (0.8197ⴕ) |
|  | **Model 5** | **<0.01** | 0.8806 (0.8496ⴕ) | 0.052 | 0.9008 (0.8703ⴕ) | **0.023** | 0.8844 (0.8534ⴕ) |
|  | **Model 6** | 0.06 | 0.9338 (0.9074ⴕ) | 0.848 | 0.9468 (0.9241ⴕ) | **0.022** | 0.9501 (0.9236ⴕ) |
|  | **Model 7** | 0.094 | 0.9735 (0.9535ⴕ) | 0.516 | 0.9744 (0.9512ⴕ) | 0.075 | 0.971 (0.9527ⴕ) |
|  | **Model 8** | 0.071 | 0.7999 (0.7569ⴕ) | 0.41 | 0.8008 (0.7485ⴕ) | 0.062 | 0.7967 (0.7464ⴕ) |
| **Models** | | **MiR-21-5p + MiR-151a-3p** | | **MiR-122-5p + MiR-126-5p** | | **MiR-21-5p + MiR-122-5p** | |
|  |  | **P-value** | **AUROC** | **P-value** | **AUROC** | **P-value** | **AUROC** |
| **Univariate** | | **0.037** | 0.6955 (0.6842ⴕ) | **0.021** | 0.6947 (0.6796ⴕ) | 0.095 | 0.6717 (0.6591ⴕ) |
| **Multivariate (model 1 and others contributing variables)** | **Model 1** | **0.032** | 0.7657 (0.7171ⴕ) | **0.02** | 0.7783 (0.7365ⴕ) | 0.102 | 0.7639 (0.728ⴕ) |
|  | **Model 2** | 0.052 | 0.9201 (0.8946ⴕ) | 0.100 | 0.9241 (0.8978ⴕ) | 0.591 | 0.9116 (0.889ⴕ) |
|  | **Model 3** | **<0.01** | 0.8885 (0.8555ⴕ) | **<0.01** | 0.8907 (0.8578ⴕ) | 0.035 | 0.8657 (0.829ⴕ) |
|  | **Model 4** | **<0.01** | 0.8534 (0.8099ⴕ) | **<0.01** | 0.8632 (0.8251ⴕ) | 0.073 | 0.835 (0.7912) |
|  | **Model 5** | **<0.01** | 0.8806 (0.8491ⴕ) | **<0.01** | 0.8894 (0.8611ⴕ) | 0.049 | 0.8603 (0.8207ⴕ) |
|  | **Model 6** | 0.06 | 0.9338 (0.9063ⴕ) | **<0.01** | 0.9493 (0.9263ⴕ) | 0.142 | 0.9271 (0.9023ⴕ) |
|  | **Model 7** | 0.094 | 0.9735 (0.9563ⴕ) | 0.064 | 0.971 (0.9519ⴕ) | 0.418 | 0.966 (0.948ⴕ) |
|  | **Model 8** | 0.071 | 0.7999 (0.7507ⴕ) | **0.034** | 0.7993 (0.7536ⴕ) | 0.237 | 0.787 (0.7322ⴕ) |

Abbreviations: AUROC, Area under the Receiver Operating Characteristic Curve; miR, microRNA; ⴕ Optimism corrected AUROC value. P-value for the miRNAs in the logistic regression model. Models are adjusted. Model 1: adjusted by sex, age, physical activity. Model 2: adjusted by sex, age, physical activity and adiponectin. Model 3: adjusted by sex, age, physical activity and RBP4 (Retinol Binding Protein 4). Model 4: adjusted by sex, age, physical activity and triglycerides. Model 5: adjusted by sex, age, physical activity and glucose. Model 6: adjusted by sex, age, physical activity and LECT2 (Leukocyte cell-derived chemotaxin-2). Model 7: adjusted by sex, age, physical activity and leptin. Model 8: adjusted by sex, age, physical activity and chemerin.

**Supplementary Table 6**. Logistic regressions analyses between hepatic fat content and combinations of miRNAs.

| **Models** | | **Hepatic fat content (%)** | | | | | |
| --- | --- | --- | --- | --- | --- | --- | --- |
|  |  | **MiR-151a-3p + MiR-122-5p** | | **MiR-151a-3p + MiR-126-5p** | | **MiR-21-5p + MiR-126-5p** | |
|  |  | **P-value** | **AUROC** | **P-value** | **AUROC** | **P-value** | **AUROC** |
| **Univariate** | | 0.202 | 0.6561 (0.631ⴕ) | 0.061 | 0.6322 (0.6074ⴕ) | **0.023** | 0.6384 (0.6172ⴕ) |
| **Multivariate (model 1 and others contributing variables)** | **Model 1** | 0.273 | 0,756 (0.7065ⴕ) | 0.1287 | 0.7581 (0.7129ⴕ) | 0.073 | 0.7649 (0.7257ⴕ) |
|  | **Model 2** | 0.432 | 0.8489 (0.8081ⴕ) | 0.17 | 0.8502 (0.8113ⴕ) | 0.175 | 0.8469 (0.8124ⴕ) |
|  | **Model 3** | 0.126 | 0.8346 (0.7905ⴕ) | **0.036** | 0.8459 (0.8034ⴕ) | **0.023** | 0.8457 (0.8119ⴕ) |
|  | **Model 4** | 0.116 | 0.8363 (0.794ⴕ) | 0.069 | 0.8363 (0.7921ⴕ) | 0.076 | 0.8224 (0.7765ⴕ) |
|  | **Model 5** | 0.052 | 0.8563 (0.8198ⴕ) | 0.062 | 0.8554 (0.8179ⴕ) | 0.087 | 0.8469 (0.8108ⴕ) |
|  | **Model 6** | 0.544 | 0.8545 (0.8217ⴕ) | 0.457 | 0.8554 (0.8166ⴕ) | 0.199 | 0.8621 (0.8321ⴕ) |
|  | **Model 7** | 0.343 | 0.8422 (0.8095ⴕ) | 0.165 | 0.8493 (0.8097ⴕ) | 0.073 | 0.8546 (0.8192ⴕ) |
|  | **Model 8** | 0.493 | 0.7807 (0.7305ⴕ) | 0.237 | 0.7842 (0.7289ⴕ) | 0.152 | 0.7898 (0.7422ⴕ) |
| **Models** | | **MiR-21-5p + MiR-151a-3p** | | **MiR-122-5p + MiR-126-5p** | | **MiR-21-5p + MiR-122-5p** | |
|  |  | **P-value** | **AUROC** | **P-value** | **AUROC** | **P-value** | **AUROC** |
| **Univariate** | | 0.744 | 0.6561 (0.6348ⴕ) | 0.994 | 0.6395 (0.6132ⴕ) | 0.102 | 0.6397 (0.6164ⴕ) |
| **Multivariate (model 1 and others contributing variables)** | **Model 1** | 0.773 | 0.7664 (0.7177ⴕ) | 0.862 | 0.7611 (0.7205ⴕ) | 0.142 | 0.7693 (0.7217ⴕ) |
|  | **Model 2** | 0.941 | 0.8493 (0.8053ⴕ) | 0.415 | 0.8406 (0.8026ⴕ) | 0.435 | 0.8455 (0.8078ⴕ) |
|  | **Model 3** | 0.82 | 0.8389 (0.8016ⴕ) | 0.539 | 0.8287 (0.7838ⴕ) | 0.082 | 0.8421 (0.798ⴕ) |
|  | **Model 4** | 0.796 | 0.8233 (0.7774ⴕ) | 0.979 | 0.8172 (0.7664ⴕ) | 0.114 | 0.8226 (0.7786ⴕ) |
|  | **Model 5** | 0.478 | 0.8532 (0.8167ⴕ) | 0.795 | 0.8389 (0.7961ⴕ) | 0.073 | 0.8530 (0.8118ⴕ) |
|  | **Model 6** | 0.422 | 0.8531 (0.8162ⴕ) | 0.876 | 0.8476 (0.8075ⴕ) | 0.197 | 0.8584 (0.8242ⴕ) |
|  | **Model 7** | 0.705 | 0,8426 (0.8043ⴕ) | 0.517 | 0.8438 (0.8034ⴕ) | 0.166 | 0.8440 (0.8044ⴕ) |
|  | **Model 8** | 0.744 | 0.7781 (0.7268ⴕ) | 0.633 | 0.7853 (0.7297ⴕ) | 0.309 | 0.7876 (0.7412ⴕ) |

Abbreviations: AUROC, Area under the Receiver Operating Characteristic Curve; miR, microRNA; ⴕ Optimism corrected AUROC value. P-value for the miRNAs in the logistic regression model. Models are adjusted. Model 1: adjusted by sex, age, physical activity. Model 2: adjusted by sex, age, physical activity and adiponectin. Model 3: adjusted by sex, age, physical activity and RBP4 (Retinol Binding Protein 4). Model 4: adjusted by sex, age, physical activity and triglycerides. Model 5: adjusted by sex, age, physical activity and glucose. Model 6: adjusted by sex, age, physical activity and LECT2 (Leukocyte cell-derived chemotaxin-2). Model 7 : adjusted by sex, age, physical activity and leptin. Model 8: adjusted by sex, age, physical activity and chemerin.

**Supplementary Table 7**. Selected miRNAs and their validated target genes (mRNAs) related to fatty liver disease according to public databases (miRWalk and DisGeNet).

| **miRNAs** | **Validated Target genes (mRNA)** |
| --- | --- |
| MiR-122-5p | *ADAM10, ADM2, ANXA11, ARSA, CCNG1, CDK4, CREB1, DNAJB1, F2RL1, FANCC, GALNT10, GFPT1, GLUL, GP2, MAGT1, MAPK1, MGAT1, MOGAT3, NCAM1, NMNAT2, NOD2, NPEPPS, PARVB, PEG10, POFUT1, PTPN2, RBM38, SLC15A2, SLC31A1, SLC52A2, TNFSF14, TRIB1, UBE2K, UBE2L3, USP10, USP28, VHL, WNT1, YME1L1* |
| MiR-151a-3p | *AZIN2, EMB, HADH, MCL1, YWHAQ* |
| MiR-126-5p | *CD55, CDCA5, ECE1, EHD3, EPHA4, HADHA, HYKK, MOB1A, MYLK, OPRD1, P2RY1, PCYT1A, PLXNA3, PRKAA2, PTGER3, SEC63, SORT1, TRDMT1, YAP1* |
| MiR-21-5p | *CCR7, CLOCK, DDAH1, DNM1L, EIF4EBP2, FMR1, HOXA9, NT5C2, SOX5, ZNF217.* |

Abbrevations: miR, microRNA; mRNA,

**Supplementary Table 8**. Biological processes and metabolic pathways from validated target genes associated with miR-151a-3p.

| **Metabolic pathways** | **Genes**  **found** | **Input**  **size** | **Term**  **genes** | **Universe** | **Pval** | **Pval_adj** | **Genes** |
| --- | --- | --- | --- | --- | --- | --- | --- |
| PI3K-Akt signaling pathway | 2 | 4 | 353 | 7806 | 0.01151 | 0.0673 | *MCL1, YWHAQ* |
| Fatty acid elongation | 1 | 4 | 27 | 7806 | 0.0137 | 0.0673 | *HADH* |
| Butanoate metabolism | 1 | 4 | 28 | 7806 | 0.0142 | 0.0673 | *HADH* |
| Tryptophan metabolism | 1 | 4 | 42 | 7806 | 0.0213 | 0.0673 | *HADH* |
| Fatty acid degradation | 1 | 4 | 43 | 7806 | 0.0218 | 0.0673 | *HADH* |
| Valine, leucine and isoleucine degradation | 1 | 4 | 48 | 7806 | 0.0243 | 0.0673 | *HADH* |
| Arginine and proline metabolism | 1 | 4 | 50 | 7806 | 0.0253 | 0.0673 | *AZIN2* |
| Fatty acid metabolism | 1 | 4 | 57 | 7806 | 0.0288 | 0.0673 | *HADH* |
| Lysine degradation | 1 | 4 | 63 | 7806 | 0.0319 | 0.0673 | *HADH* |
| Cell cycle | 1 | 4 | 126 | 7806 | 0.0630 | 0.0955 | *YWHAQ* |
| Oocyte meiosis | 1 | 4 | 131 | 7806 | 0.0654 | 0.0955 | *YWHAQ* |
| Apoptosis | 1 | 4 | 136 | 7806 | 0.0679 | 0.0955 | *MCL1* |
| Hippo signaling pathway | 1 | 4 | 157 | 7806 | 0.0780 | 0.0955 | *YWHAQ* |
| Hepatitis C | 1 | 4 | 157 | 7806 | 0.0780 | 0.0955 | *YWHAQ* |
| Hepatitis B | 1 | 4 | 162 | 7806 | 0.0804 | 0.0955 | *YWHAQ* |
| JAK-STAT signaling pathway | 1 | 4 | 162 | 7806 | 0.0804 | 0.0955 | *MCL1* |
| Viral carcinogenesis | 1 | 4 | 201 | 7806 | 0.099 | 0.1107 | *YWHAQ* |
| MicroRNAs in cancer | 1 | 4 | 216 | 7806 | 0.1061 | 0.112 | *MCL1* |
| Metabolic pathways | 2 | 4 | 1516 | 7806 | 0.1719 | 0.1719 | *AZIN2, HADH* |

**Supplementary Table 9**. Biological processes and metabolic pathways from validated target genes associated with miR-122-5p.

| **Metabolic pathways** | **Genes**  **found** | **Input**  **size** | **Termgenes** | **Universe** | **Pval** | **Pval_adj** | **Genes** |
| --- | --- | --- | --- | --- | --- | --- | --- |
| Cushing syndrome | 4 | 28 | 155 | 7806 | 0.0021 | 0.2704 | *CREB1, MAPK1, CDK4, WNT1* |
| Melanogenesis | 3 | 28 | 101 | 7806 | 0.0054 | 0.2704 | *CREB1, MAPK1, WNT1* |
| TNF signaling pathways | 3 | 28 | 112 | 7806 | 0.0072 | 0.2704 | *CREB1, NOD2, MAPK1* |
| Alanine, aspartate and glutamate metabolism | 2 | 28 | 37 | 7806 | 0.0076 | 0.2704 | *GLUL, GFPT1* |
| Bladder cancer | 2 | 28 | 41 | 7806 | 0.0093 | 0.2704 | *MAPK1, CDK4* |
| Other types of O-glycan biosynthesis | 2 | 28 | 47 | 7806 | 0.0121 | 0.2704 | *POFUT1, GALNT10* |
| Ubiquitin mediated proteolysis | 3 | 28 | 142 | 7806 | 0.0138 | 0.2704 | *VHL, UBE2K, UBE2L3* |
| Breast cancer | 3 | 28 | 147 | 7806 | 0.0151 | 0.2704 | *MAPK1, CDK4, WNT1* |
| Hepatocellular carcinoma | 3 | 28 | 168 | 7806 | 0.0216 | 0.2704 | *MAPK1, CDK4, WNT1* |
| Influenza A | 3 | 28 | 171 | 7806 | 0.0226 | 0.2704 | *MAPK1, DNAJB1, CDK4* |
| Renal cell carcinoma | 2 | 28 | 69 | 7806 | 0.0251 | 0.2704 | *VHL, MAPK1* |
| Tuberculosis | 3 | 28 | 179 | 7806 | 0.0255 | 0.2704 | *CREB1, NOD2, MAPK1* |
| Melanoma | 2 | 28 | 72 | 7806 | 0.0271 | 0.2704 | *MAPK1, CDK4* |
| Non-small cell lung cancer | 2 | 28 | 72 | 7806 | 0.0271 | 0.2704 | *MAPK1, CDK4* |
| p53 signaling pathway | 2 | 28 | 73 | 7806 | 0.0278 | 0.2704 | *CCNG1, CDK4* |
| Platinum drug resistance | 2 | 28 | 73 | 7806 | 0.0278 | 0.2704 | *MAPK1, SLC31A1* |
| Human papillomavirus infection | 4 | 28 | 331 | 7806 | 0.0290 | 0.2704 | *CREB1, MAPK1, CDK4, WNT1* |
| Glioma | 2 | 28 | 75 | 7806 | 0.0293 | 0.2704 | *MAPK1, CDK4* |
| Chronic myeloid leukemia | 2 | 28 | 76 | 7806 | 0.03003 | 0.2704 | *MAPK1, CDK4* |
| Pancreatic cancer | 2 | 28 | 76 | 7806 | 0.03003 | 0.2704 | *MAPK1, CDK4* |
| Kaposi sarcoma-associated herpesvirus infection | 3 | 28 | 194 | 7806 | 0.03137 | 0.2704 | *CREB1, MAPK1, CDK4* |
| Viral carcinogenesis | 3 | 28 | 201 | 7806 | 0.0343 | 0.2825 | *CREB1, MAPK1, CDK4* |
| Chemical carcinogenesis - receptor activation | 3 | 28 | 208 | 7806 | 0.0374 | 0.2947 | *CREB1, MAPK1, WNT1* |
| Human T-cell leukemia virus 1 infection | 3 | 28 | 222 | 7806 | 0.0440 | 0.3029 | *CREB1, MAPK1, CDK4* |
| Human cytomegalovirus infection | 3 | 28 | 223 | 7806 | 0.0445 | 0.3029 | *CREB1, MAPK1, CDK4* |
| Endocrine resistance | 2 | 28 | 96 | 7806 | 0.0459 | 0.3029 | *MAPK1, CDK4* |
| Circadian entrainment | 2 | 28 | 97 | 7806 | 0.0468 | 0.3029 | *CREB1, MAPK1* |
| Prostate cancer | 2 | 28 | 97 | 7806 | 0.0468 | 0.3029 | *CREB1, MAPK1* |
| AGE-RAGE signaling pathway in diabetic complications | 2 | 28 | 100 | 7806 | 0.0494 | 0.3089 | *MAPK1, CDK4* |
| T cell receptor signaling pathway | 2 | 28 | 104 | 7806 | 0.053 | 0.3140 | *MAPK1, CDK4* |
| Parathyroid hormone synthesis, secretion and action | 2 | 28 | 106 | 7806 | 0.0549 | 0.3140 | *CREB1, MAPK1* |
| Insulin resistance | 2 | 28 | 108 | 7806 | 0.0567 | 0.3140 | *CREB1, GFPT1* |
| HIF-1 signaling pathway | 2 | 28 | 109 | 7806 | 0.0577 | 0.3140 | *VHL, MAPK1* |
| Nitrogen metabolism | 1 | 28 | 17 | 7806 | 0.0593 | 0.3140 | *GLUL* |
| Cholinergic synapse | 2 | 28 | 113 | 7806 | 0.0615 | 0.3140 | *CREB1, MAPK1* |
| Glutamatergic synapse | 2 | 28 | 114 | 7806 | 0.0624 | 0.3140 | *GLUL, MAPK1* |
| Prion disease | 3 | 28 | 260 | 7806 | 0.0648 | 0.3173 | *CREB1, NCAM1, MAPK1* |
| Growth hormone synthesis, secretion and action | 2 | 28 | 119 | 7806 | 0.0673 | 0.3208 | *CREB1, MAPK1* |
| Osteoclast differentiation | 2 | 28 | 127 | 7806 | 0.0754 | 0.3421 | *CREB1, MAPK1* |
| Arginine biosynthesis | 1 | 28 | 22 | 7806 | 0.0761 | 0.3421 | *GLUL* |
| Relaxin signaling pathway | 2 | 28 | 129 | 7806 | 0.0775 | 0.3421 | *CREB1, MAPK1* |
| Vascular smooth muscle contraction | 2 | 28 | 134 | 7806 | 0.0827 | 0.3566 | *ADM2, MAPK1* |
| Estrogen signaling pathway | 2 | 28 | 137 | 7806 | 0.08595 | 0.3618 | *CREB1, MAPK1* |
| Signaling pathways regulating pluripotency of stem cells | 2 | 28 | 143 | 7806 | 0.0924 | 0.3763 | *MAPK1, WNT1* |
| Gastric cancer | 2 | 28 | 149 | 7806 | 0.0991 | 0.3763 | *MAPK1, WNT1* |
| Adrenergic signaling in cardiomyocytes | 2 | 28 | 150 | 7806 | 0.1002 | 0.3763 | *CREB1, MAPK1* |
| Glyoxylate and dicarboxylate metabolism | 1 | 28 | 30 | 7806 | 0.1023 | 0.3763 | *GLUL* |
| mTOR signaling pathway | 2 | 28 | 154 | 7806 | 0.1047 | 0.3763 | *MAPK1, WNT1* |
| Circadian rhythm | 1 | 28 | 31 | 7806 | 0.1056 | 0.3763 | *CREB1* |
| Cellular senescence | 2 | 28 | 156 | 7806 | 0.107 | 0.3763 | *MAPK1, CDK4* |
| Hepatitis C | 2 | 28 | 157 | 7806 | 0.1082 | 0.3763 | *MAPK1, CDK4* |
| Hepatitis B | 2 | 28 | 162 | 7806 | 0.114 | 0.3763 | *CREB1, MAPK1* |
| Nicotinate and nicotinamide metabolism | 1 | 28 | 35 | 7806 | 0.1184 | 0.3763 | *NMNAT2* |
| Pathways in cancer | 4 | 28 | 531 | 7806 | 0.1189 | 0.3763 | *VHL, MAPK1, CDK4, WNT1* |
| cGMP-PKG signaling pathway | 2 | 28 | 167 | 7806 | 0.1198 | 0.3763 | *CREB1, MAPK1* |
| African trypanosomiasis | 1 | 28 | 36 | 7806 | 0.1216 | 0.3763 | *F2RL1* |
| Mucin type O-glycan biosynthesis | 1 | 28 | 36 | 7806 | 0.1216 | 0.3763 | *GALNT10* |
| Aldosterone-regulated sodium reabsorption | 1 | 28 | 37 | 7806 | 0.1247 | 0.3763 | *MAPK1* |
| Biosynthesis of nucleotide sugars | 1 | 28 | 37 | 7806 | 0.1247 | 0.3763 | *GFPT1* |
| Thyroid cancer | 1 | 28 | 37 | 7806 | 0.1247 | 0.3763 | *MAPK1* |
| PI3K-Akt signaling pathway | 3 | 28 | 353 | 7806 | 0.1309 | 0.3826 | *CREB1, MAPK1, CDK4* |
| Various types of N-glycan biosynthesis | 1 | 28 | 39 | 7806 | 0.131 | 0.3826 | *MGAT1* |
| Alcoholism | 2 | 28 | 183 | 7806 | 0.1391 | 0.3935 | *CREB1, MAPK1* |
| NOD-like receptor signaling pathway | 2 | 28 | 183 | 7806 | 0.1391 | 0.3935 | *NOD2, MAPK1* |
| Fat digestion and absorption | 1 | 28 | 43 | 7806 | 0.1435 | 0.3960 | *MOGAT3* |
| Alzheimer disease | 3 | 28 | 371 | 7806 | 0.1458 | 0.3960 | *ADAM10, MAPK1, WNT1* |
| Vasopressin-regulated water reabsorption | 1 | 28 | 44 | 7806 | 0.1466 | 0.3960 | *CREB1* |
| Type II diabetes mellitus | 1 | 28 | 46 | 7806 | 0.1527 | 0.4066 | *MAPK1* |
| Focal adhesion | 2 | 28 | 201 | 7806 | 0.1616 | 0.4070 | *MAPK1, PARVB* |
| Amino sugar and nucleotide sugar metabolism | 1 | 28 | 49 | 7806 | 0.1619 | 0.4070 | *GFPT1* |
| Cocaine addiction | 1 | 28 | 49 | 7806 | 0.1619 | 0.4070 | *CREB1* |
| Sphingolipid metabolism | 1 | 28 | 49 | 7806 | 0.1619 | 0.4070 | *ARSA* |
| N-Glycan biosynthesis | 1 | 28 | 50 | 7806 | 0.1649 | 0.4077 | *MGAT1* |
| Proteoglycans in cancer | 2 | 28 | 205 | 7806 | 0.1667 | 0.4077 | *MAPK1, WNT1* |
| Fanconi anemia pathway | 1 | 28 | 54 | 7806 | 0.1769 | 0.4269 | *FANCC* |
| MicroRNAs in cancer | 2 | 28 | 216 | 7806 | 0.1808 | 0.4299 | *CCNG1, MAPK1* |
| cAMP signaling pathway | 2 | 28 | 221 | 7806 | 0.1873 | 0.4299 | *CREB1, MAPK1* |
| Endometrial cancer | 1 | 28 | 58 | 7806 | 0.1887 | 0.4299 | *MAPK1* |
| VEGF signaling pathway | 1 | 28 | 59 | 7806 | 0.1916 | 0.4299 | *MAPK1* |
| Long-term depression | 1 | 28 | 60 | 7806 | 0.1946 | 0.4299 | *MAPK1* |
| Mineral absorption | 1 | 28 | 60 | 7806 | 0.1946 | 0.4299 | *SLC31A1* |
| Glycerolipid metabolism | 1 | 28 | 61 | 7806 | 0.1975 | 0.4299 | *MOGAT3* |
| Basal cell carcinoma | 1 | 28 | 63 | 7806 | 0.2033 | 0.4299 | *WNT1* |
| GnRH secretion | 1 | 28 | 64 | 7806 | 0.2061 | 0.4299 | *MAPK1* |
| Cortisol synthesis and secretion | 1 | 28 | 65 | 7806 | 0.209 | 0.4299 | *CREB1* |
| Inflammatory bowel disease | 1 | 28 | 65 | 7806 | 0.209 | 0.4299 | *NOD2* |
| Acute myeloid leukemia | 1 | 28 | 67 | 7806 | 0.2147 | 0.4299 | *MAPK1* |
| Fc epsilon RI signaling pathway | 1 | 28 | 67 | 7806 | 0.2147 | 0.4299 | *MAPK1* |
| Long-term potentiation | 1 | 28 | 67 | 7806 | 0.2147 | 0.4299 | *MAPK1* |
| Amphetamine addiction | 1 | 28 | 69 | 7806 | 0.2204 | 0.4299 | *CREB1* |
| Renin secretion | 1 | 28 | 69 | 7806 | 0.2204 | 0.4299 | *CREB1* |
| Central carbon metabolism in cancer | 1 | 28 | 70 | 7806 | 0.2232 | 0.4299 | *MAPK1* |
| Epithelial cell signaling in Helicobacter pylori infection | 1 | 28 | 70 | 7806 | 0.2232 | 0.4299 | *ADAM10* |
| Prolactin signaling pathway | 1 | 28 | 70 | 7806 | 0.2232 | 0.4299 | *MAPK1* |
| Adherens junction | 1 | 28 | 71 | 7806 | 0.226 | 0.4307 | *MAPK1* |
| Pathways of neurodegeneration - multiple diseases | 3 | 28 | 463 | 7806 | 0.2294 | 0.4325 | *MAPK1, UBE2L3, WNT1* |
| Biosynthesis of amino acids | 1 | 28 | 74 | 7806 | 0.2344 | 0.4343 | *GLUL* |
| Thyroid hormone synthesis | 1 | 28 | 75 | 7806 | 0.2372 | 0.4343 | *CREB1* |
| Leishmaniasis | 1 | 28 | 76 | 7806 | 0.2399 | 0.4343 | *MAPK1* |
| Pertussis | 1 | 28 | 76 | 7806 | 0.2399 | 0.4343 | *MAPK1* |
| Antigen processing and presentation | 1 | 28 | 78 | 7806 | 0.2454 | 0.4399 | *CREB1* |
| EGFR tyrosine kinase inhibitor resistance | 1 | 28 | 79 | 7806 | 0.2482 | 0.4404 | *MAPK1* |
| B cell receptor signaling pathway | 1 | 28 | 80 | 7806 | 0.2509 | 0.4409 | *MAPK1* |
| ErbB signaling pathway | 1 | 28 | 85 | 7806 | 0.2644 | 0.4463 | *MAPK1* |
| Colorectal cancer | 1 | 28 | 86 | 7806 | 0.267 | 0.4463 | *MAPK1* |
| Insulin secretion | 1 | 28 | 86 | 7806 | 0.267 | 0.4463 | *CREB1* |
| Gap junction | 1 | 28 | 88 | 7806 | 0.2723 | 0.4463 | *MAPK1* |
| GABAergic synapse | 1 | 28 | 89 | 7806 | 0.275 | 0.4463 | *GLUL* |
| Longevity regulating pathway | 1 | 28 | 89 | 7806 | 0.275 | 0.4463 | *CREB1* |
| PD-L1 expression and PD-1 checkpoint pathway in cancer | 1 | 28 | 89 | 7806 | 0.275 | 0.4463 | *MAPK1* |
| Small cell lung cancer | 1 | 28 | 92 | 7806 | 0.2829 | 0.4463 | *CDK4* |
| Th1 and Th2 cell differentiation | 1 | 28 | 92 | 7806 | 0.2829 | 0.4463 | *MAPK1* |
| GnRH signaling pathway | 1 | 28 | 93 | 7806 | 0.2855 | 0.4463 | *MAPK1* |
| IL-17 signaling pathway | 1 | 28 | 94 | 7806 | 0.288 | 0.4463 | *MAPK1* |
| TGF-beta signaling pathway | 1 | 28 | 94 | 7806 | 0.288 | 0.4463 | *MAPK1* |
| Fc gamma R-mediated phagocytosis | 1 | 28 | 96 | 7806 | 0.2932 | 0.4463 | *MAPK1* |
| Metabolic pathways | 7 | 28 | 1516 | 7806 | 0.2933 | 0.4463 | *MGAT1, GLUL, MOGAT3, ARSA, NMNAT2, GFPT1, GALNT10* |
| Aldosterone synthesis and secretion | 1 | 28 | 98 | 7806 | 0.2983 | 0.4463 | *CREB1* |
| Choline metabolism in cancer | 1 | 28 | 98 | 7806 | 0.2983 | 0.4463 | *MAPK1* |
| Inflammatory mediator regulation of TRP channels | 1 | 28 | 98 | 7806 | 0.2983 | 0.4463 | *F2RL1* |
| Viral protein interaction with cytokine and cytokine receptor | 1 | 28 | 98 | 7806 | 0.2983 | 0.4463 | *TNFSF14* |
| Chagas disease | 1 | 28 | 101 | 7806 | 0.306 | 0.4467 | *MAPK1* |
| NF-kappa B signaling pathway | 1 | 28 | 102 | 7806 | 0.3085 | 0.4467 | *TNFSF14* |
| Progesterone-mediated oocyte maturation | 1 | 28 | 102 | 7806 | 0.3085 | 0.4467 | *MAPK1* |
| Toll-like receptor signaling pathway | 1 | 28 | 102 | 7806 | 0.3085 | 0.4467 | *MAPK1* |
| C-type lectin receptor signaling pathway | 1 | 28 | 104 | 7806 | 0.3135 | 0.4504 | *MAPK1* |
| Glucagon signaling pathway | 1 | 28 | 106 | 7806 | 0.3185 | 0.4539 | *CREB1* |
| Th17 cell differentiation | 1 | 28 | 108 | 7806 | 0.323 | 0.4574 | *MAPK1* |
| Toxoplasmosis | 1 | 28 | 112 | 7806 | 0.3332 | 0.4673 | *MAPK1* |
| Serotonergic synapse | 1 | 28 | 113 | 7806 | 0.3356 | 0.4673 | *MAPK1* |
| Neurotrophin signaling pathway | 1 | 28 | 119 | 7806 | 0.35 | 0.4792 | *MAPK1* |
| Sphingolipid signaling pathway | 1 | 28 | 119 | 7806 | 0.35 | 0.4792 | *MAPK1* |
| AMPK signaling pathway | 1 | 28 | 120 | 7806 | 0.3524 | 0.4792 | *CREB1* |
| Thyroid hormone signaling pathway | 1 | 28 | 121 | 7806 | 0.3547 | 0.4792 | *MAPK1* |
| Platelet activation | 1 | 28 | 124 | 7806 | 0.3618 | 0.4819 | *MAPK1* |
| Neuroactive ligand-receptor interaction | 2 | 28 | 352 | 7806 | 0.3621 | 0.4819 | *ADM2, F2RL1* |
| Cell cycle | 1 | 28 | 126 | 7806 | 0.3664 | 0.4841 | *CDK4* |
| Natural killer cell mediated cytotoxicity | 1 | 28 | 130 | 7806 | 0.3756 | 0.4846 | *MAPK1* |
| FoxO signaling pathway | 1 | 28 | 131 | 7806 | 0.3779 | 0.4846 | *MAPK1* |
| Oocyte meiosis | 1 | 28 | 131 | 7806 | 0.3779 | 0.4846 | *MAPK1* |
| Dopaminergic synapse | 1 | 28 | 132 | 7806 | 0.3802 | 0.4846 | *CREB1* |
| Lysosome | 1 | 28 | 132 | 7806 | 0.3802 | 0.4846 | *ARSA* |
| Apoptosis | 1 | 28 | 136 | 7806 | 0.3892 | 0.4862 | *MAPK1* |
| Yersinia infection | 1 | 28 | 136 | 7806 | 0.3892 | 0.4862 | *MAPK1* |
| Insulin signaling pathway | 1 | 28 | 137 | 7806 | 0.3914 | 0.4862 | *MAPK1* |
| Apelin signaling pathway | 1 | 28 | 139 | 7806 | 0.3958 | 0.4862 | *MAPK1* |
| Measles | 1 | 28 | 139 | 7806 | 0.3958 | 0.4862 | *CDK4* |
| Autophagy - animal | 1 | 28 | 141 | 7806 | 0.4002 | 0.4862 | *MAPK1* |
| Retrograde endocannabinoid signaling | 1 | 28 | 141 | 7806 | 0.4002 | 0.4862 | *MAPK1* |
| Phospholipase D signaling pathway | 1 | 28 | 147 | 7806 | 0.4133 | 0.4979 | *MAPK1* |
| Cell adhesion molecules | 1 | 28 | 148 | 7806 | 0.4154 | 0.4979 | *NCAM1* |
| Biosynthesis of cofactors | 1 | 28 | 153 | 7806 | 0.426 | 0.5065 | *NMNAT2* |
| Oxytocin signaling pathway | 1 | 28 | 154 | 7806 | 0.4281 | 0.5065 | *MAPK1* |
| Hippo signaling pathway | 1 | 28 | 157 | 7806 | 0.4344 | 0.5097 | *WNT1* |
| Necroptosis | 1 | 28 | 158 | 7806 | 0.4364 | 0.5097 | *GLUL* |
| JAK-STAT signaling pathway | 1 | 28 | 162 | 7806 | 0.4446 | 0.5159 | *PTPN2* |
| Wnt signaling pathway | 1 | 28 | 167 | 7806 | 0.4547 | 0.5243 | *WNT1* |
| Tight junction | 1 | 28 | 169 | 7806 | 0.4587 | 0.5245 | *CDK4* |
| Protein processing in endoplasmic reticulum | 1 | 28 | 170 | 7806 | 0.4607 | 0.5245 | *DNAJB1* |
| Axon guidance | 1 | 28 | 182 | 7806 | 0.484 | 0.5475 | *MAPK1* |
| Neutrophil extracellular trap formation | 1 | 28 | 185 | 7806 | 0.4897 | 0.5505 | *MAPK1* |
| Chemokine signaling pathway | 1 | 28 | 190 | 7806 | 0.4990 | 0.5541 | *MAPK1* |
| Diabetic cardiomyopathy | 1 | 28 | 190 | 7806 | 0.4990 | 0.5541 | *GFPT1* |
| Pathogenic Escherichia coli infection | 1 | 28 | 196 | 7806 | 0.5099 | 0.5628 | *MAPK1* |
| Epstein-Barr virus infection | 1 | 28 | 201 | 7806 | 0.5189 | 0.5692 | *CDK4* |
| Chemical carcinogenesis - reactive oxygen species | 1 | 28 | 210 | 7806 | 0.5346 | 0.5778 | *MAPK1* |
| Rap1 signaling pathway | 1 | 28 | 210 | 7806 | 0.5346 | 0.5778 | *MAPK1* |
| Human immunodeficiency virus 1 infection | 1 | 28 | 211 | 7806 | 0.53631 | 0.577 | *MAPK1* |
| Lipid and atherosclerosis | 1 | 28 | 214 | 7806 | 0.5414 | 0.5799 | *MAPK1* |
| Regulation of actin cytoskeleton | 1 | 28 | 218 | 7806 | 0.5481 | 0.582 | *MAPK1* |
| Thermogenesis | 1 | 28 | 219 | 7806 | 0.5498 | 0.582 | *CREB1* |
| Coronavirus disease - COVID-19 | 1 | 28 | 230 | 7806 | 0.5678 | 0.5973 | *MAPK1* |
| Ras signaling pathway | 1 | 28 | 232 | 7806 | 0.5709 | 0.5973 | *MAPK1* |
| Shigellosis | 1 | 28 | 247 | 7806 | 0.5941 | 0.6176 | *MAPK1* |
| Salmonella infection | 1 | 28 | 249 | 7806 | 0.5971 | 0.6176 | *MAPK1* |
| Parkinson disease | 1 | 28 | 253 | 7806 | 0.6031 | 0.6202 | *UBE2L3* |
| Cytokine-cytokine receptor interaction | 1 | 28 | 293 | 7806 | 0.6580 | 0.6666 | *TNFSF14* |
| Huntington disease | 1 | 28 | 293 | 7806 | 0.6580 | 0.6666 | *CREB1* |
| MAPK- signaling pathway | 1 | 28 | 294 | 7806 | 0.6593 | 0.6666 | *MAPK1* |
| Amyotrophic lateral sclerosis | 1 | 28 | 351 | 7806 | 0.7248 | 0.7288 | *ANXA11* |
| Herpes simplex virus 1 infection | 1 | 28 | 494 | 7806 | 0.8401 | 0.8401 | *TNFSF14* |

**Supplementary Table 10**. Biological processes and metabolic pathways from validated target genes associated with miR-21-5p.

| **Metabolic pathways** | **Genesfound** | **Input**  **size** | **Term**  **genes** | **Universe** | **Pval** | **Pval_adj** | **Genes** |
| --- | --- | --- | --- | --- | --- | --- | --- |
| Circadian rhythm | 1 | 6 | 31 | 7806 | 0.0235 | 0.1611 | *CLOCK* |
| Nicotinate and nicotinamide metabolism | 1 | 6 | 35 | 7806 | 0.0266 | 0.1611 | *NT5C2* |
| Pyrimidine metabolism | 1 | 6 | 58 | 7806 | 0.0437 | 0.1611 | *NT5C2* |
| Longevity regulating pathway - multiple species | 1 | 6 | 62 | 7806 | 0.0467 | 0.1611 | *EIF4EBP2* |
| Viral protein interaction with cytokine and cytokine receptor | 1 | 6 | 98 | 7806 | 0.073 | 0.1611 | *CCR7* |
| TNF signaling pathway | 1 | 6 | 112 | 7806 | 0.083 | 0.1611 | *DNM1L* |
| Purine metabolism | 1 | 6 | 126 | 7806 | 0.093 | 0.1611 | *NT5C2* |
| Dopaminergic synapse | 1 | 6 | 132 | 7806 | 0.0972 | 0.1611 | *CLOCK* |
| Necroptosis | 1 | 6 | 158 | 7806 | 0.1154 | 0.1611 | *DNM1L* |
| NOD-like receptor signaling pathway | 1 | 6 | 183 | 7806 | 0.1327 | 0.1611 | *DNM1L* |
| Chemokine signaling pathway | 1 | 6 | 190 | 7806 | 0.1374 | 0.1611 | *CCR7* |
| Transcriptional misregulation in cancer | 1 | 6 | 191 | 7806 | 0.1381 | 0.1611 | *HOXA9* |
| Cytokine-cytokine receptor interaction | 1 | 6 | 293 | 7806 | 0.2051 | 0.2209 | *CCR7* |
| Metabolic pathways | 1 | 6 | 1516 | 7806 | 0.7263 | 0.7263 | *NT5C2* |

**Supplementary Table 11**. Biological processes and metabolic pathways from validated target genes associated with miR-126-5p.

| **Metabolic pathways** | **Genes found** | **Input size** | **Term genes** | **Universe** | **Pval** | **Pval_adj** | **Genes** |
| --- | --- | --- | --- | --- | --- | --- | --- |
| Hippo signaling pathway - multiple species | 2 | 17 | 29 | 7806 | 0.0017 | 0.1068 | *YAP1, MOB1A* |
| Lysine degradation | 2 | 17 | 63 | 7806 | 0.008 | 0.24598 | *HYKK, HADHA* |
| Phosphonate and phosphinate metabolism | 1 | 17 | 6 | 7806 | 0.013 | 0.2574 | *PCYT1A* |
| Platelet activation | 2 | 17 | 124 | 7806 | 0.0291 | 0.2574 | *P2RY1, MYLK* |
| Apelin signaling pathway | 2 | 17 | 139 | 7806 | 0.0359 | 0.2574 | *MYLK, PRKAA2* |
| Neuroactive ligand-receptor interaction | 3 | 17 | 352 | 7806 | 0.0386 | 0.2574 | *P2RY1, PTGER3, OPRD1* |
| Oxytocin signaling pathway | 2 | 17 | 154 | 7806 | 0.0433 | 0.2574 | *MYLK, PRKAA2* |
| Hippo signaling pathway | 2 | 17 | 157 | 7806 | 0.0448 | 0.2574 | *YAP1, MOB1A* |
| Protein export | 1 | 17 | 23 | 7806 | 0.0489 | 0.2574 | *SEC63* |
| cGMP-PKG signaling pathway | 2 | 17 | 167 | 7806 | 0.0501 | 0.2574 | *OPRD1, MYLK* |
| Fatty acid elongation | 1 | 17 | 27 | 7806 | 0.0572 | 0.2574 | *HADHA* |
| Axon guidance | 2 | 17 | 182 | 7806 | 0.0584 | 0.2574 | *EPHA4, PLXNA3* |
| Butanoate metabolism | 1 | 17 | 28 | 7806 | 0.0593 | 0.2574 | *HADHA* |
| beta-Alanine metabolism | 1 | 17 | 31 | 7806 | 0.0654 | 0.2574 | *HADHA* |
| Circadian rhythm | 1 | 17 | 31 | 7806 | 0.0654 | 0.2574 | *PRKAA2* |
| Propanoate metabolism | 1 | 17 | 32 | 7806 | 0.0675 | 0.2574 | *HADHA* |
| Tryptophan metabolism | 1 | 17 | 42 | 7806 | 0.0877 | 0.2994 | *HADHA* |
| Fatty acid degradation | 1 | 17 | 43 | 7806 | 0.0897 | 0.2994 | *HADHA* |
| Calcium signaling pathway | 2 | 17 | 238 | 7806 | 0.0932 | 0.2994 | *PTGER3, MYLK* |
| Valine, leucine and isoleucine degradation | 1 | 17 | 48 | 7806 | 0.0996 | 0.3008 | *HADHA* |
| Cholesterol metabolism | 1 | 17 | 50 | 7806 | 0.10351 | 0.3008 | *SORT1* |
| Regulation of lipolysis in adipocytes | 1 | 17 | 55 | 7806 | 0.1133 | 0.3078 | *PTGER3* |
| Fatty acid metabolism | 1 | 17 | 57 | 7806 | 0.1172 | 0.3078 | *HADHA* |
| Viral myocarditis | 1 | 17 | 59 | 7806 | 0.1211 | 0.3078 | *CD55* |
| Longevity regulating pathway - multiple species | 1 | 17 | 62 | 7806 | 0.1268 | 0.3096 | *PRKAA2* |
| Adipocytokine signaling pathway | 1 | 17 | 69 | 7806 | 0.14023 | 0.329 | *PRKAA2* |
| Gastric acid secretion | 1 | 17 | 76 | 7806 | 0.1533 | 0.3465 | *MYLK* |
| Complement and coagulation cascades | 1 | 17 | 85 | 7806 | 0.1699 | 0.3470 | *CD55* |
| Taste transduction | 1 | 17 | 85 | 7806 | 0.1699 | 0.3470 | *P2RY1* |
| Longevity regulating pathway | 1 | 17 | 89 | 7806 | 0.1772 | 0.3470 | *PRKAA2* |
| Hypertrophic cardiomyopathy | 1 | 17 | 90 | 7806 | 0.179 | 0.3470 | *PRKAA2* |
| Glycerophospholipid metabolism | 1 | 17 | 98 | 7806 | 0.1934 | 0.3470 | *PCYT1A* |
| Choline metabolism in cancer | 1 | 17 | 98 | 7806 | 0.1934 | 0.3470 | *PCYT1A* |
| Hematopoietic cell lineage | 1 | 17 | 98 | 7806 | 0.1934 | 0.3470 | *CD55* |
| Glucagon signaling pathway | 1 | 17 | 106 | 7806 | 0.2075 | 0.3558 | *PRKAA2* |
| Insulin resistance | 1 | 17 | 108 | 7806 | 0.211 | 0.3558 | *PRKAA2* |
| Sphingolipid signaling pathway | 1 | 17 | 119 | 7806 | 0.23 | 0.3558 | *OPRD1* |
| Neurotrophin signaling pathway | 1 | 17 | 119 | 7806 | 0.23 | 0.3558 | *SORT1* |
| AMPK signaling pathway | 1 | 17 | 120 | 7806 | 0.23172 | 0.3558 | *PRKAA2* |
| FoxO signaling pathway | 1 | 17 | 131 | 7806 | 0.2502 | 0.3558 | *PRKAA2* |
| Lysosome | 1 | 17 | 132 | 7806 | 0.2519 | 0.3558 | *SORT1* |
| Vascular smooth muscle contraction | 1 | 17 | 134 | 7806 | 0.2552 | 0.3558 | *MYLK* |
| Insulin signaling pathway | 1 | 17 | 137 | 7806 | 0.2601 | 0.3558 | *PRKAA2* |
| Fluid shear stress and atherosclerosis | 1 | 17 | 139 | 7806 | 0.2634 | 0.3558 | *PRKAA2* |
| Autophagy - animal | 1 | 17 | 141 | 7806 | 0.2666 | 0.3558 | *PRKAA2* |
| Alcoholic liver disease | 1 | 17 | 142 | 7806 | 0.2683 | 0.3558 | *PRKAA2* |
| Non-alcoholic fatty liver disease | 1 | 17 | 151 | 7806 | 0.2828 | 0.3654 | *PRKAA2* |
| mTOR signaling pathway | 1 | 17 | 154 | 7806 | 0.2875 | 0.3654 | *PRKAA2* |
| Tight junction | 1 | 17 | 169 | 7806 | 0.3109 | 0.3812 | *PRKAA2* |
| Protein processing in endoplasmic reticulum | 1 | 17 | 170 | 7806 | 0.3125 | 0.3812 | *SEC63* |
| Focal adhesion | 1 | 17 | 201 | 7806 | 0.3584 | 0.4166 | *MYLK* |
| Rap1 signaling pathway | 1 | 17 | 210 | 7806 | 0.3712 | 0.4166 | *P2RY1* |
| MicroRNAs in cancer | 1 | 17 | 216 | 7806 | 0.3796 | 0.4166 | *CDCA5* |
| Regulation of actin cytoskeleton | 1 | 17 | 218 | 7806 | 0.3824 | 0.4166 | *MYLK* |
| Thermogenesis | 1 | 17 | 219 | 7806 | 0.3838 | 0.4166 | *PRKAA2* |
| cAMP signaling pathway | 1 | 17 | 221 | 7806 | 0.3866 | 0.4166 | *PTGER3* |
| Human cytomegalovirus infection | 1 | 17 | 223 | 7806 | 0.3893 | 0.4166 | *PTGER3* |
| Endocytosis | 1 | 17 | 251 | 7806 | 0.4266 | 0.4486 | *EHD3* |
| PI3K-Akt signaling pathway | 1 | 17 | 353 | 7806 | 0.545 | 0.5635 | *PRKAA2* |
| Metabolic pathways | 3 | 17 | 1516 | 7806 | 0.6694 | 0.6805 | *HYKK, PCYT1A, HADHA* |
| Pathways in cancer | 1 | 17 | 531 | 7806 | 0.6984 | 0.6984 | *PTGER3* |

**Supplementary Figure 2.** Pathway enrichment of putative candidates of miR-122-5p (**A**), miR-151a-3p (B), miR-126-5p (**C**), and miR-21-5p (**D**). The top 10 metabolic pathways according to p-adjusted value were depicted for the target genes associated with each miRNA.


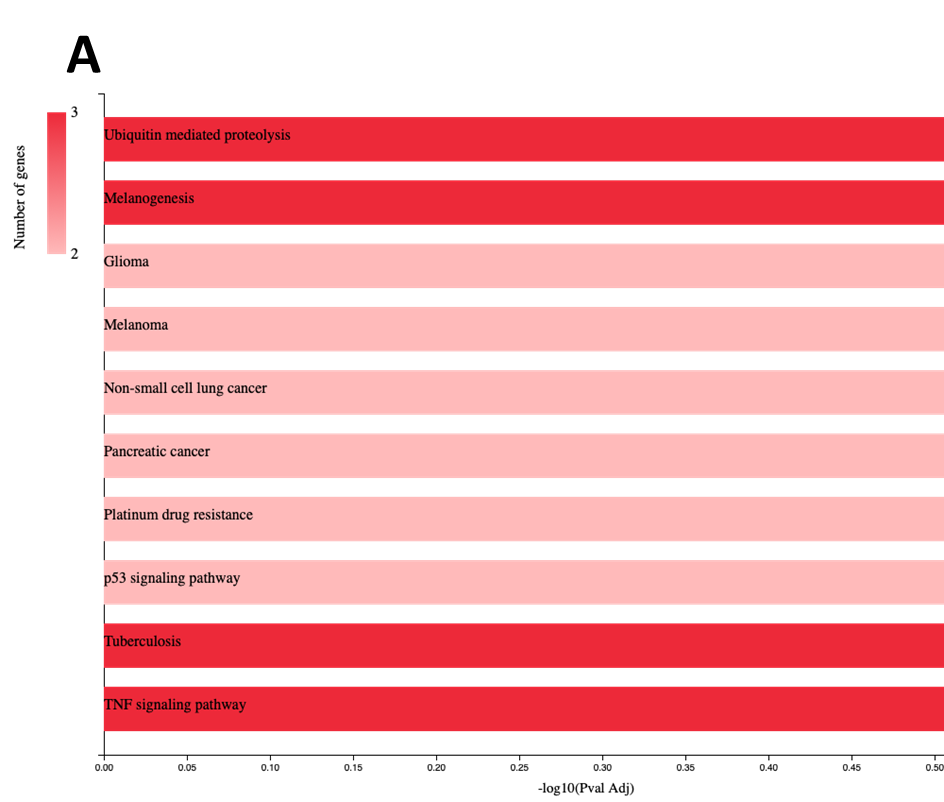


**
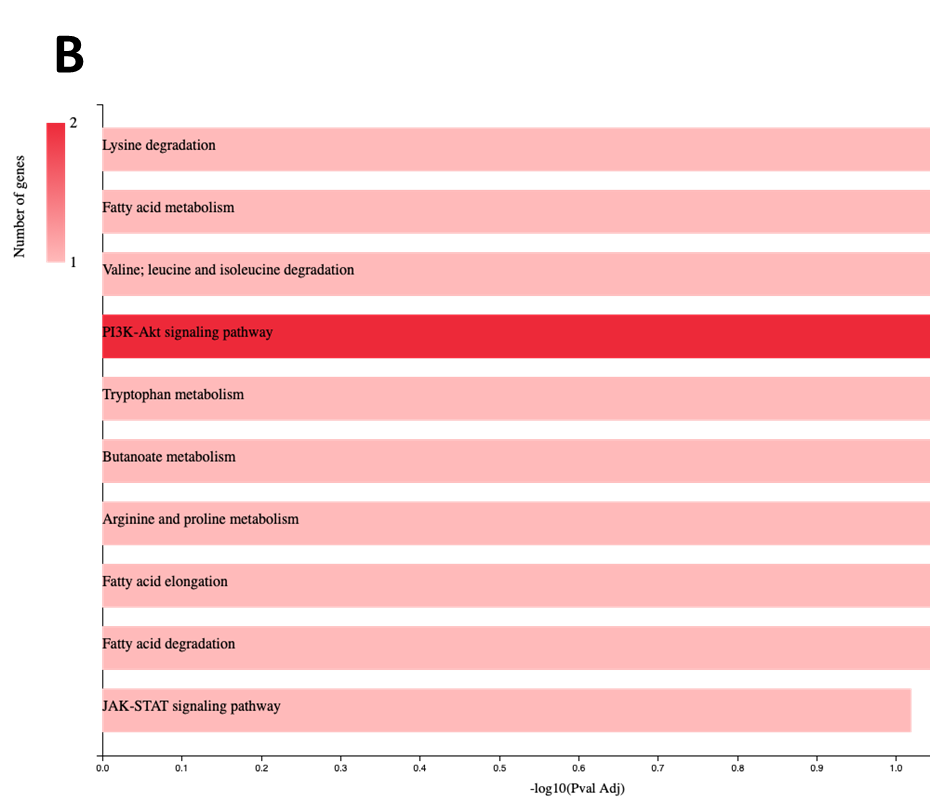
**

**
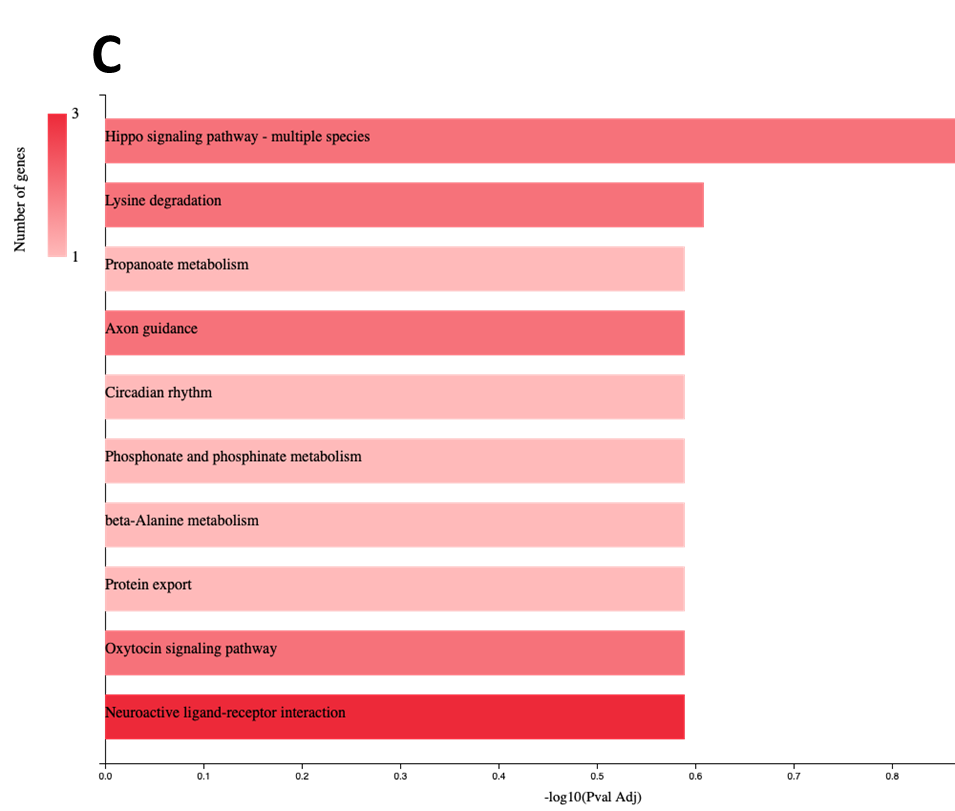
**

**
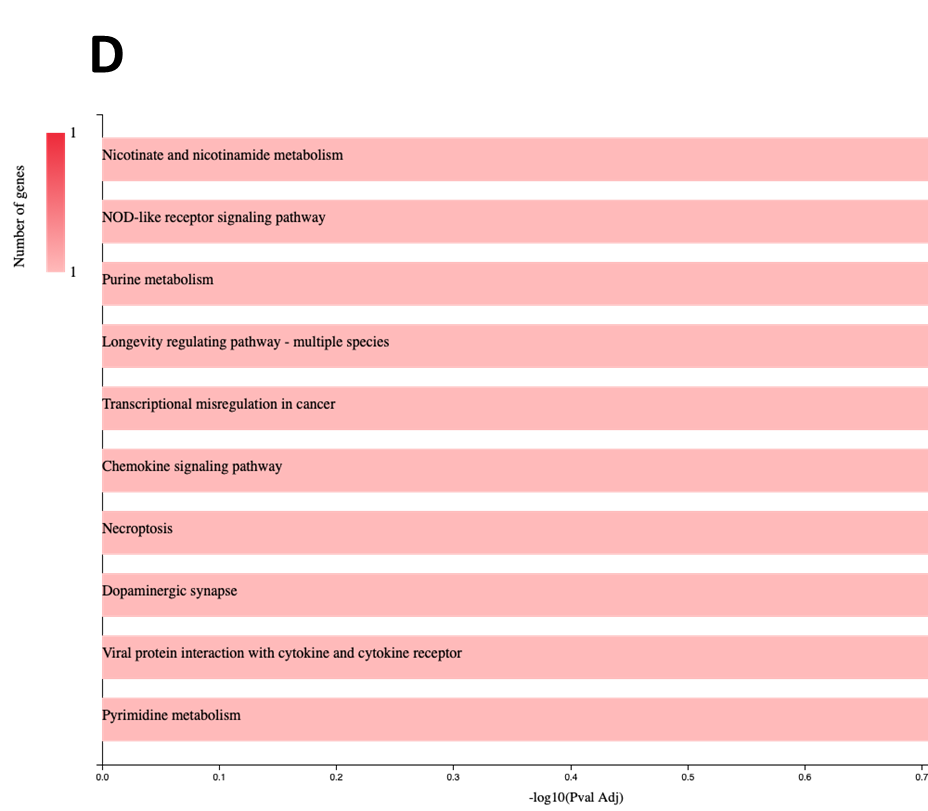
**

**
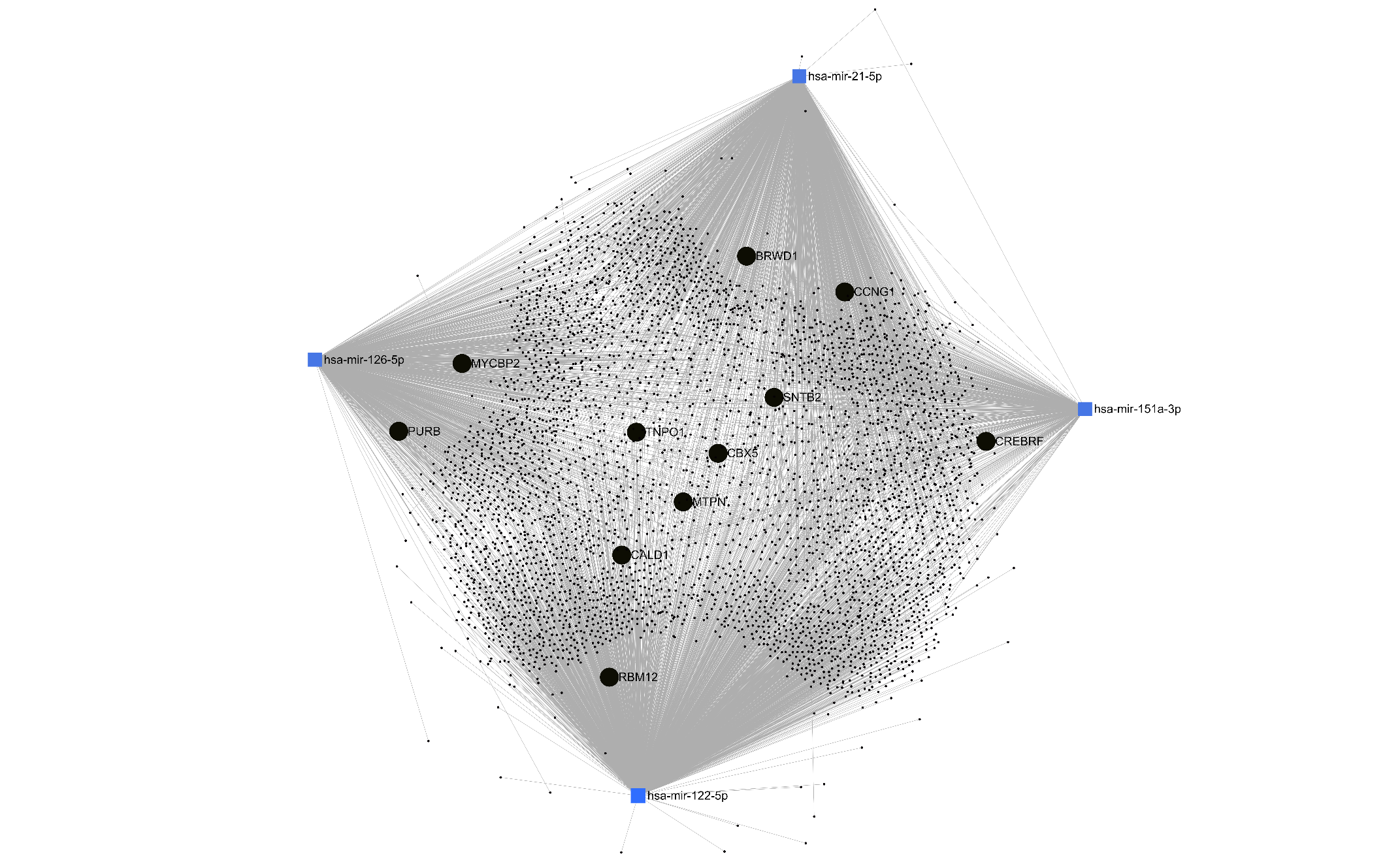
**

**Supplementary Figure 3.** Network of target genes and our specific miRNAs. Genes shared between miR-122-5p, miR-151a-3p, miR-126-5p, and miR-21-5p are amplified and highlighted in black, while these miRNAs are marked in blue. Target genes were obtained through miRTaBase v8.0 and TarBase v8.0, choosing *homo sapiens* as a main organism (ID type miRbase).
